# Neutrophil-Specific Knockdown of β2 Integrins Impairs Antifungal Effector Functions and Aggravates the Course of Invasive Pulmonal Aspergillosis

**DOI:** 10.3389/fimmu.2022.823121

**Published:** 2022-06-06

**Authors:** Maximilian Haist, Frederic Ries, Matthias Gunzer, Monika Bednarczyk, Ekkehard Siegel, Michael Kuske, Stephan Grabbe, Markus Radsak, Matthias Bros, Daniel Teschner

**Affiliations:** ^1^Department of Dermatology, University Medical Center of the Johannes Gutenberg University, Mainz, Germany; ^2^Department of Hematology, Medical Oncology and Pneumology, University Medical Center of the Johannes Gutenberg University, Mainz, Germany; ^3^Institute for Experimental Immunology and Imaging, University Hospital, University Duisburg-Essen, Essen, Germany; ^4^Leibniz-Institut für Analytische Wissenschaften ISAS -e.V, Dortmund, Germany; ^5^Institute for Medical Microbiology and Hygiene, University Medical Center of the Johannes Gutenberg University, Mainz, Germany; ^6^Department of Internal Medicine II, University Hospital Würzburg, Würzburg, Germany

**Keywords:** β2 integrins, CD18, CD11b, polymorphonuclear neutrophils, *Aspergillus fumigatus*, pneumonia, complement receptor 3, phagocytosis

## Abstract

β2-integrins are heterodimeric surface receptors that are expressed specifically by leukocytes and consist of a variable α (CD11a-d) and a common β-subunit (CD18). Functional impairment of CD18, which causes leukocyte adhesion deficiency type-1 results in an immunocompromised state characterized by severe infections, such as invasive pulmonary aspergillosis (IPA). The underlying immune defects have largely been attributed to an impaired migratory and phagocytic activity of polymorphonuclear granulocytes (PMN). However, the exact contribution of β2-integrins for PMN functions *in-vivo* has not been elucidated yet, since the mouse models available so far display a constitutive CD18 knockout (CD18^-/-^ or CD18^hypo^). To determine the PMN-specific role of β2-integrins for innate effector functions and pathogen control, we generated a mouse line with a Ly6G-specific knockdown of the common β-subunit (CD18^Ly6G^ cKO). We characterized CD18^Ly6G^ cKO mice *in-vitro* to confirm the PMN-specific knockdown of β2-integrins. Next, we investigated the clinical course of IPA in *A. fumigatus* infected CD18^Ly6G^ cKO mice with regard to the fungal burden, pulmonary inflammation and PMN response towards *A. fumigatus*. Our results revealed that the β2-integrin knockdown was restricted to PMN and that CD18^Ly6G^ cKO mice showed an aggravated course of IPA. In accordance, we observed a higher fungal burden and lower levels of proinflammatory innate cytokines, such as TNF-α, in lungs of IPA-infected CD18^Ly6G^ cKO mice. Bronchoalveolar lavage revealed higher levels of CXCL1, a stronger PMN-infiltration, but concomitantly elevated apoptosis of PMN in lungs of CD18^Ly6G^ cKO mice. E*x-vivo* analysis further unveiled a strong impairment of PMN effector function, as reflected by an attenuated phagocytic activity, and a diminished generation of reactive oxygen species (ROS) and neutrophil-extracellular traps (NET) in CD18-deficient PMN. Overall, our study demonstrates that β2-integrins are required specifically for PMN effector functions and contribute to the clearance of *A. fumigatus* by infiltrating PMN, and the establishment of an inflammatory microenvironment in infected lungs.

## 1 Introduction

Humans are constantly exposed to spores of the ubiquitous environmental mould *Aspergillus fumigatus* (*A. fumigatus*) (1, 2). Although *A. fumigatus* is usually well controlled in healthy individuals, *A. fumigatus* can cause lethal invasive pulmonary aspergillosis (IPA) in immunocompromised patients, e.g., due to chemotherapeutic treatment of malignant diseases or immunosuppressive therapy after allogeneic hematopoietic stem cell transplantation, with mortality varying between 30% and 90% (1, 3). Commonly, disease follows the inhalation of airborne conidia, which germinate in the lung of immunocompromised hosts, sprouting there as hyphae (4). Despite the clinical application of potent antifungal drugs for prophylaxis and treatment of invasive fungal diseases in patients with severe immune deficiency, IPA continues to be a highly relevant health issue in daily clinical care (5).

The small size of *A. fumigatus* conidia (2-3µm) allows them to bypass the physiological epithelial defence of the nasal and bronchial cavities and to reach the lung alveoli without being cleared by the ciliated bronchial epithelium (6, 7). Although several *in-vitro* studies indicated that epithelial cells may internalize and subject conidia to phagolysosomal degradation (8), an engulfment of conidia by bronchial epithelium has not been observed *in-vivo* so far (9). Hence, the clearance of *A. fumigatus* conidia requires effective cellular and humoral immune responses.

The innate immune system is considered the key player in the clearance of conidia and the defence against the outgrowth of *A. fumigatus* conidia. Here, resident leukocytes present in the alveolar lung tissue, such as alveolar macrophages and dendritic cells (DC) initiate an early response against invasive aspergillosis (10, 11). However, the recruitment of polymorphonuclear neutrophils (PMN) to the lung tissue is essential for an efficient clearing of *A. fumigatus* (5, 6, 12). The importance of PMN for an effective protection against IPA was inferred from the observation, that quantitative [i.e., in neutropenic patients (13)] or qualitative [i.e., patients with chronic granulomatous disease (14)] defects of PMN are critical predisposing factors for IPA (13, 15). PMN mediate the killing of *A. fumigatus via* different effector mechanisms dependent on the size of conidia and hyphae:

Since the size of hyphae prevents phagocytosis, hyphal killing is mainly conferred by oxidative and non-oxidative PMN effector functions. These include the generation of reactive oxygen species (ROS), the formation of neutrophil extracellular traps (NET) and the release of neutrophil granular content (6, 16, 17). In the context of oxidative PMN functions, it has been observed, that the common beta subunit of β2 integrins (CD18) is critical for the recognition of *A. fumigatus* and the subsequent generation of ROS (18, 19).

By contrast, the small-size of *A. fumigatus* conidia allows for the phagocytosis by PMN, which is either mediated by direct recognition *via* complement receptor 3 (CR3, i.e., CD11b/CD18), Dectin-1 or indirectly *via* complement-dependant opsonization (6, 18, 19). The importance of β2 integrins in PMN-functions has been confirmed in more recent reports, which revealed that an antibody-mediated blockade of CD11b prevents the generation of ROS (20) and phagocytosis of *A. fumigatus* conidia by PMN (21).

The ß2 integrin-family consists of four members, which are formed by heterodimerization of the common beta subunit (CD18) with a variable alpha subunit (CD11a-CD11d) (22, 23). The integrin receptor CR-3 is primarily expressed by leukocytes of the myeloid lineage, which was name-giving (macrophage antigen 1, MAC-1) (22). MAC-1 serves as an adhesion receptor for various ligands, including intercellular adhesion molecule 1 (ICAM-1), which is necessary for the transendothelial migration of macrophages and PMN (5, 24). MAC1/CR3 also binds complement-opsonized pathogens, and immune complexes, non-opsonized pathogens, and numerous serum factors (25). In addition, MAC-1 serves as a coreceptor for the Fc-receptor-mediated uptake of antibody-opsonized pathogens (26). It has further been shown that MAC-1 acts as a regulator of LPS-induced signaling in macrophages and DC, and that the engagement of MAC-1 with yet unrecognized T cell receptors mediates T cell activation (27, 28). Last, MAC-1 is a modifier of various signaling pathways (29), such as TLR-induced inflammatory signaling (27), which is involved in the innate immune response to invasive aspergillosis (10).

In accordance with the importance of β2 integrins for immune responses, loss-of-function mutations of the CD18 gene in humans result in the so-called leukocyte adhesion deficiency type 1 (LAD1) syndrome, being characterized by severe, recurrent bacterial and fungal infections in patients, which require extensive treatment with anti-infective agents (30). Several studies have indicated that an impaired migration and phagocytic activity of CD18-deficient PMN might be largely causative for the spreading of pathogens in LAD1 patients (31).

However, the exact contribution of β2 integrins for PMN functions *in-vivo* has not been fully elucidated yet, since the mouse models available so far either display a constitutive CD18-knockdown (CD18^hypo^) or knockout (CD18^-/-^), which complicates to delineate the cell-type specific role of CD18. In order to reveal the PMN-specific role of β2-integrins for the control of infectious diseases such as IPA, we established a transgenic mouse with a floxed CD18 gene (CD18^fl/fl^ Ly6G^Cre-^). By crossing CD18^fl/fl^ mice with transgenic mice expressing *Cre* recombinase under control of the PMN-specific (Ly6G^Cre+^) promoter, offspring with a PMN-specific knockdown of CD18 have been generated, thus allowing to analyze the PMN-specific role in IPA.

In this study, we show that mice with a Ly6G-specific knockdown of CD18 (CD18^Ly6G^ conditional knockout, in the following termed CD18^Ly6G^ cKO) display an impaired survival during IPA as compared to control-mice (CD18^fl/fl^). The impaired survival of CD18^Ly6G^ cKO mice is reflected by a higher fungal burden in the lung of these mice during the early phase of pulmonary infection and lower amounts of proinflammatory innate mediators, such as TNF-α in the bronchoalveolar lavage fluid (BALF). By contrast, we detected an enhanced bronchial infiltration of PMN and elevated levels of the PMN-chemoattractant CXCL-1 in BALF derived from infected CD18^Ly6G^ cKO mice, which might reflect a compensatory mechanism. Moreover, we could observe that CD18-deficient PMN showed a strong attenuation of effector functions *in-vitro*, which might explain the higher fungal burden in the lungs of infected CD18^Ly6G^ cKO mice. In particular, we observed an impaired phagocytic uptake of *A. fumigatus* conidia, and a diminished generation of ROS and NET in CD18-deficient PMN.

## 2 Materials and Methods

### 2.1 Fungal Strains and Cultivation Conditions

The wild type (WT; ATCC 46645) and the GFP-modified (AfS148) *A. fumigatus* strains (32) were cultured in Aspergillus minimal medium (AMM) with 1% (w/v) glucose, 1% Hutner´s trace element solution and 1M MgSO_4_ (Carl Roth, Karlsruhe, Germany) as described earlier (16). Briefly, conidia were incubated on AMM agar plates for 4 days at 37°C and 5% CO_2_. For preparation of spore suspensions, plates were washed with sterile water containing a small amount of glass pearls (Ø 4mm; Carl Roth, Karlsruhe, Germany) to detach conidia from agar plates. The obtained spore suspension was filtered twice through a sterile 40 μm nylon mesh and stored in sterile water at 4°C.

### 2.2 Mice

In order to allow for the assessment of the importance of β2-integrins specifically for PMN, we generated a transgenic mouse strain with a floxed CD18 gene (CD18^fl/fl^ Ly6G^Cre-^ ; B6.Cg-Itgb2^tm2.GrabS^), which enabled a conditional knockout of β2 integrins in a cell-type specific manner (**Supplementary Figure 1**). The generation of mice with floxed exon 3 of the CD18 gene locus will be described in detail elsewhere. CD18^fl/fl^ mice were bred with transgenic mice expressing *Cre* recombinase under control of the PMN-specific *Ly6G* promoter (33, 34) as described by Hasenberg and coworkers (Ly6G^Cre+^, C57BL/6-*Ly6g*(tm2621(Cre-tdTomato)Arte mice) (35), yielding a mouse strain with diminished levels of CD18 on neutrophils (CD18^Ly6G^ cKO). Resulting CD18^wt/fl^ Ly6G^Cre-^ offspring were crossed back to CD18^fl/fl^ background. Derived male CD18^fl/fl^ Ly6G^Cre-^ mice were paired with CD18^fl/fl^ Ly6GCre- females, yielding mice with diminished levels of CD18 on neutrophils (CD18^fl/fl^ Ly6G^Cre-^, in the following termed CD18Ly6G cKO) and CD18^fl/fl^ Ly6G^Cre-^ mice at the same ratio.

The mouse strains (CD18^fl/fl^ Ly6G^Cre-^ and CD18^Ly6G^ cKO) were maintained in the Translational Animal Research Center of the University Medical Center Mainz under pathogen-free conditions on a standard diet. All animal procedures were performed in accordance with the institutional guidelines and approved by the responsible national authority (National Investigation Office Rhineland-Pfalz, Approval ID: 23177-07/G16-1-020). For the experiments, mice of both sexes were used, although most experiments were done with female mice. Mice used in the experiments were aged between 6-18 weeks unless stated otherwise.

### 2.3 Mouse Genotyping

Gene-targeted animals were verified by PCR (**Supplementary Figure 1**). To this end, ear biopsies of mice (2–6 weeks) were incubated with lysis buffer containing 100µl Direct PCR Ear Buffer (Viagen Biotec, Los Angeles, CA, USA) and 2µl proteinase K (ThermoFisher Scientific, Waltham, MA). Samples were incubated at 56°C for 1-3h under shaking. Subsequently the suspension was heated to 95°C for 5 min to inactivate proteinase K, and the lysate was put on ice until further processing. The typical PCR reaction contained a 25-μl volume containing 5µl PCR Reaction Mix (Sigma Aldrich, Merck, Darmstadt, Germany), 17,3µl H_2_O, 0,2µl of myTaq-Polymerase (Roche, Mannheim, Germany) and 1µl of the primers (10pmol/µl) for PCR 1 (Mix of 2 primers: CD18 ex3_s2, B2(s): 5´-GTGACACTTTAC TTGCGACCA-3´; CD18 loxp_as1,B3(as): 5´-TGCCAATAAAGAATTTCAGAGCC-3´, suspended 1:10 in H_2_O) or for PCR 2 (Mix of 3 primers: Ly6G [78]-s for 5´-CCTGCAACCTGGTCAGAGAG-3´, and 5064_61_rev for 5´-GAGGTCCAAGAGACTTTCTGG-3´, and 2240_31 for 5′-ACGTCCAGACACAGCATAGG-3′ suspended 1:10 in H_2_O). In PCR 2 we also included a control pair of primers for amplifying Actin as a wild-type allele (Actin FW: 5´-TGTTACCAACTGGGACGACA-3´ and Actin REV: 5´-GACATGCAAGGAGTGCAAGA. The following PCR conditions were applied for PCR 1:initial denaturation (3 min, 95°C), followed by 35 cycles (denaturation: 30 s, 95°C; annealing: 30 s, 58°C; elongation: 45 s, 72°C) and by a final elongation step (2 min 72°C). For PCR 2 the following PCR conditions were applied: initial denaturation (5 min, 95°C), followed by 35 cycles (denaturation: 30 s, 95°C; annealing: 30 s, 60°C; elongation: 1 min, 72°C) and by a final elongation step (10 min 72°C). PCR products were analyzed by agarose gel electrophoresis (**Supplementary Figure 1**).

### 2.4 Mouse Model of Invasive Aspergillosis

Mice were anesthetized with 14.5% Ketamin (50mg/ml)/5.7% Xylazin (0.2%) and were subsequently challenged with 10^7^ *A. fumigatus* conidia (strain ATCC 46645) applied intratracheally as described (35, 36). In brief, a 22G indwelling venous catheter (Vasofix, B. Braun AG, Melsungen, Germany) was inserted into the trachea and 100 μl sterile fungal suspension was administered through the catheter. To enhance dispersion in the lungs, mice were ventilated mechanically with 250 breaths/min, 300 μl/breath for 2 min using an animal respirator (MiniVent, Hugo Sachs, March-Hugstetten, Germany) as previously described (16). In order to characterize the early immune response to fungal infection, 10 mice/group were sacrificed 24h after infection. In two additional groups (n=5-8 mice/group) the course of systemic infection was daily examined by evaluation of weight, activity, breathing, overall appearance (as assessed by posture, skin, and fur appearance), and survival was monitored for 14 days. Mice with severe symptoms as determined by clinical scoring were immediately euthanized as required by the institutional animal ethics guidelines. Where indicated, PMN depletion was induced by i.p. injection of anti-Gr-1 antibody (150 μg, clone RB6-8C5; BioXCell, Lebanon, NH) 1 day prior to inoculation with fungal suspension.

### 2.5 Flow Cytometric Analysis

Blood samples, spleens and bone marrow were prepared from sacrificed mice, and lungs were flushed with 1 ml PBS. Spleen cell suspensions were generated *via* mechanical homogenization on a 40µm nylon mesh, washed twice with cold PBS, and red blood cells (RBC) were lysed with hypotonic Gey´s solution (155mM NH_4_Cl, 10mM KHCO_3_, 10µM EDTA at pH 7,4). RBC from blood samples were lysed in the same way. Cells derived from blood, spleen, bone marrow and bronchoalveolar lavage fluid (BALF) were analyzed by flow cytometry. To this end, cells were washed with staining buffer (PBS/2% FCS), and Fc receptors were blocked by incubation with rat anti-mouse CD16/CD32 antibody (clone 2.4G2) for 15 min at 4°C. Then, cells were incubated with FITC-conjugated anti-CD86 (GL-1), anti-CD45 (30F11), and anti-Annexin-V (Biolegend), PerCP-conjugated anti-Ly6C (HK1.4), APC-conjugated anti-CD18 (C71/16), anti-CD14 (Sa14-2), anti-Gr-1 (RB6-8C5) and anti-CD40 (1C10), APC-eFluor 780 conjugated anti-CD11c (N418), eFluor450-conjugated anti-MHCII (M5/114 15.2) and anti-F4/80 (BM8), eFluor506-conjugated anti-CD3 (500A2), Super Bright 600-conjugated anti-CD11b (M1/70), PE-conjugated anti-CD11a (M17/4), anti-CD80 (1610A1) and anti-Ly6G (1A8), PE-eFluor610-conjugated anti-Ly6G (1A8), PE-Cyanine7-conjugated anti-CD68 (FA11) and anti-CD62L (MEL-14). All antibodies were obtained from Biolegend (San Diego, CA) or Thermo Fisher (Waltham, MA). Viability was assessed using Fixable-viability-dye (FVD), conjugated either with APC eFluor 780, eFluor 450 or eFluor 506 (ThermoFisher). Samples were analyzed using a flow cytometer (Attune™ NxT Acoustic Focusing Cytometer, Thermo Fisher), and data were processed using FlowJo software V8.8.7 (Tree Star Inc., Ashland, OR, USA). The gating strategy is shown in **Supplementary Figure 2**.

### 2.6 Quantification of Fungal Burden

The right lungs of euthanized mice were removed, mechanically homogenized and serial dilutions were plated on Sabouraud-4% Glucose agar (Carl Roth, Karlsruhe, Germany), and cultivated at 37°C and 5% CO_2_. Colony-forming units (CFU) were counted after 24h and 48h.

Moreover, a D-Galactomannan assay based on the Platelia *Aspergillus* EIA (Bio-Rad Laboratories, Marne-La-Coquette, France) was employed to quantify the fungal load in BALF and serum derived from IPA-infected mice. This enzyme immunoassay is used in clinical routine and validated for the detection of *A. fumigatus* antigen. The test uses the rat monoclonal antibody EBA-2 directed against *Aspergillus* galactomannan. In brief, the antigen is first bound to the wells of the microplate coated with the EBA-2 antibody and then revealed by binding to the peroxidase-linked EBA-2 antibody resulting in a colorimetric reaction, which is measured *via* optical density on a Plate Reader as described previously (37).

### 2.7 Histopathologic Analysis

For histopathological analysis the left lungs of euthanized mice were filled with 10% formalin *via* the trachea. Paraffin-embedded blocks were prepared, and derived sections (5 μm) were stained with H&E to assess inflammatory responses. For this, H&E-stained sections were examined by microscopy in a blinded fashion for peribronchial, perivascular and tissue inflammation, using a scoring system (0–3). Furthermore, sections of lungs were stained with Grocott Gomori’s methenamine silver to assess the fungal burden of the lungs. Grocott stained sections were examined in a blinded fashion similar to H&E sections using a scoring system (0–3). In general, 3 randomly selected areas on each slide were analyzed with a BX40 microscope equipped with a CCD camera (Olympus, Hamburg, Germany).

### 2.8 Cytospin Analysis

For detection of lung infiltrating PMN, 100 μl of BALF containing 0.5-2x10^5^ cells (see above) were cytospun onto microscope slides (3,500 rpm for 5 min; Cytospin 3, Thermo Fisher), treated with the Diff Quick Staining Set (Microptic, Barcelona, Spain), air-dried, and fixed as recommended. Samples were analyzed using a BX50WI microscope, equipped with a CCD camera (Olympus, Hamburg, Germany). PMN were identified based on their characteristic segmented nuclei.

### 2.9 Cytokine Detection

Serum and BALF were subjected to cytokine detection by Cytometric bead array (CBA) using the mouse CBA flex sets following the manufacturer’s instructions (BD Bioscience, San Jose, CA). Similarly, *in-vitro* cytokine generation by Ly6G^+^ PMN (10^5^/100µl) immunomagnetically sorted from bone marrow of CD18^fl/fl^ and CD18^Ly6G^ cKO mice (see below) was quantified. Isolated PMN were incubated in Iscove’s medium (Thermo Fisher Scientific) supplemented with 5% (v/v) FCS, 2 mM l-glutamine, 50 μM ß-mercaptoethanol and 1 mM Na-pyruvate (SERVA Electrophoresis, Heidelberg, Germany) in 96-well plates (Greiner Bio One, Frickenhausen, Germany) and treated over-night with PBS, recombinant murine GM-CSF (100ng/ml; Miltenyi Biotec, Bergsich-Gladbach, Germany), LPS (1µg/ml, Merck-Millipore, Darmstadt, Germany), CpG (1µg/ml, *In vivo*gen, Toulouse, France) or R8/48 (1µg/ml, *In vivo*gen). Supernatants were taken 3h and 24h later from PMN aliquots generated in n=3 independent experiments.

### 2.10 Fungal Uptake by PMN

PMN were purified from bone marrow of CD18^fl/fl^ and CD18^Ly6G^ cKO mice by magnetic cell sorting (MACS) using biotin-labeled Ly6G-specific antibodies and streptavidin-conjugated beads (both from Miltenyi Biotec) according to the manufacturer’s protocol. The cell purity (Ly6G^+^) exceeded 90% as assessed by flow cytometry. Freshly isolated PMN were resuspended (10^6^ cells/ml) in cell culture medium (see above), seeded into 96-well plates (100µl/well) and were incubated with GFP-fluorescent *A. fumigatus* conidia (5) at the indicated ratios in parallel at 4°C and 37°C to differentiate mere adhesion and energy-dependent uptake. After 1h of incubation PMN were washed twice with 500µl cold PBS and stained with anti-CD11b, anti-Ly6G, anti-MHCII and anti-CD62L specific antibodies, and FVD eFluor 506 to determine the uptake and activation status of GFP-labeled conidia by flow cytometry (**Supplementary Figure 8** shows the gating strategy applied during the experiments).

### 2.11 Uptake of Inert Particles by PMN

To assess uptake of inert particles, we employed Cy5-labeled nanoparticles (Ø 50nm) and PE-labeled microBeads (Ø 2µm) (both Miltenyi Biotec). Immunomagnetically sorted PMN (10^6^ cells/ml) were incubated in cell culture medium in 96-well plates (100µl) and treated over-night (12h) with GM-CSF (100ng/ml) or LPS (1µg/ml). Subsequently, PMN were washed once with 500µl cold PBS and were either left untreated, or incubated in parallel settings with particles, and particles pre-treated with native or heat-inactivated mouse serum (hiS; 56°C, 30min) at 4°C and 37°C for various periods of time (15-60 min). Pretreatment of particles with native versus heat-inactivated mouse serum served to elucidate the complement-dependent particle uptake. Subsequently PMN were washed twice with 500µl cold PBS and incubated with anti-CD11b, anti-Ly6G, anti-MHCII, anti-CD86, anti-Ly6C and anti-CD62L antibodies and FVD eFluor 506 to determine the PMN-specific uptake of inert particles by flow cytometry.

### 2.12 Assessment of Neutrophil Apoptosis

Freshly isolated PMN (1x10^6^/ml) derived from bone marrow of either mouse strain were incubated in cell culture medium in 24-well plates and treated over-night (12h) in parallel w/o and with GM-CSF (100ng/ml), LPS (1µg/ml) and with GM-CSF plus LPS in order to differentiate spontaneous apoptosis (PBS-treated control), late-onset apoptosis (GM-CSF) and apoptosis upon LPS-treatment. Following over-night incubation, samples were washed twice with 1ml PBS and incubated with anti-Annexin V (FITC) and FVD (eFluor 506) according to the manufacturer´s protocol (ThermoFisher) to differentiate apoptosis and necrosis. Frequencies of apoptotic and necrotic PMN were determined by flow cytometry as described previously (38).

### 2.13 Analysis of ROS Production

To assess the rate of ROS production, PMN were isolated from bone marrow, were seeded into 96-well-plates (10^6^/ml; 100µl/well) washed once with 200µl PBS and resuspended in 100 μl ROS-detection solution (2 μM 2´-7´Dichlorodihydrofluorescein [DCFDA] in PBS; Alexis Biochemicals, Lausen, CHE). After 20 min of incubation at 37°C the cells were washed with 200µl PBS, centrifuged, and the sedimented cells were dispersed in 200 μl PBS. Subsequently, PMN were stimulated with GM-CSF (100ng/ml), LPS (1µg/ml), *A. fumigatus* conidia (1:1), or 100 nM PMA (Sigma-Aldrich), respectively at 37°C, 5% CO2 in triplicates. Median fluorescence intensities (MFI) were measured using a SPARK multimode microplate-reader (TECAN Trading AG, CHE) at an excitation of 485nm and an emission of 530nm for 90min (intervals of 15min). After 90min cells were analyzed by flow cytometry for DCFDA-positive events.

### 2.14 Analysis of Neutrophil-Extracellular Traps Formation

To induce the release of neutrophil extracellular traps (NET) DNA, we isolated PMN from bone marrow as described previously and seeded PMN (10^5^/100 µl) in 96-well plates with 100µl RPMI 1640 medium without phenol red (ThermoFisher, Waltham, CA). PMN were treated either with GM-CSF (100ng/ml), LPS (1µg/ml), *A. fumigatus* conidia (1:1), PMA (100 nM), or calcium ionophore (2,5µM; Sigma, Darmstadt, Germany), respectively. After incubation at 37°C for 3h, 5µM of Sytox orange nucleic stain (Invitrogen, Carlsbad, CA) was added and samples were incubated for 10min at room temperature in the dark. Subsequently, PMN were centrifugated and washed twice with 300µl cold PBS. MFI of Sytox orange was measured using a SPARK multimode microplate reader with an excitation of 547nm and an emission of 580nm. Then, cells were incubated with an anti-Ly6G antibody and analyzed by flow cytometry for Ly6G/Sytox orange double-positive cells.

### 2.15 RNA-Sequencing and Bioinformatical Analysis

First, PMN were isolated from bone marrow of CD18^fl/fl^ and CD18^Ly6G^ cKO mice (n=3). Each 10^6^ PMN were either lysed directly after isolation or cultured overnight with GM-CSF (10ng/ml) plus LPS (1µg/ml). RNA was purified with the RNeasy Plus Micro Kit according to the manufacturer’s protocol (Qiagen). RNA was quantified with a Qubit 2.0 fluorometer (Invitrogen) and the quality was assessed on a Bioanalyzer 2100 (Agilent) using a RNA 6000 Pico chip (Agilent). Samples with an RNA integrity number (RIN) of > 8 were used for library preparation. Barcoded mRNA-seq cDNA libraries were prepared from 10ng of total RNA using NEBNext^®^ Poly(A) mRNA Magnetic Isolation Module and NEBNext^®^ Ultra™ II RNA Library Prep Kit for Illumina^®^ according to the manual with a final amplification of 15 PCR cycles. Quantity was assessed using Invitrogen’s Qubit HS assay kit and library size was determined using Agilent’s 2100 Bioanalyzer HS DNA assay. Barcoded RNA-Seq libraries were onboard clustered using HiSeq^®^ Rapid SR Cluster Kit v2 using 8pM and 59bps were sequenced on the Illumina HiSeq2500 using HiSeq^®^ Rapid SBS Kit v2 (59 Cycle). The raw output data of the HiSeq was preprocessed according to the Illumina standard protocol. Sequence reads were trimmed for adapter sequences and further processed using Qiagen’s software CLC Genomics Workbench (v20.0 with CLC’s default settings for RNA-Seq analysis). Reads were aligned to GRCm38 genome. Sequencing data were first analyzed with CLC Genomics Work Bench (Qiagen). Further processing was performed in R using the DESeq2 package for calling differential gene expression (39, 40). To determine the most up- or downregulated genes, genes were sorted on the basis of log_2_ [fold change] maximum-likelihood estimation, and the *P*-value cut-off was set to 0.05. Results were illustrated using the pheatmap package. Functional interaction networks were visualized using the STRING package in the open-source platform Cytoscape.

### 2.16 Statistical Analysis

Statistical analysis was conducted with GraphPad Prism (version 5.0a; GraphPad Software, San Diego, CA, USA). Comparison of two different parameters was performed using paired Student’s *t*-test. In case of comparison of more than two groups we employed one-way ANOVA and posthoc Tukey test. For survival analysis, Kaplan-Meier plots and hazard ratios have been calculated. For all analyses, p < 0.05 was considered as statistically significant. Abbreviations: *p<0.05, **p<0.005, ***p<0,001.

## 3 Results

### 3.1 Phenotype and Impairment of PMN Effector Functions of CD18^Ly6G^ cKO Mice Assessed by *In-Vitro* Experiments

In murine leukocytes Ly6G is selectively expressed by PMN (41). To obtain mice with a diminished CD18 expression specifically on PMN (CD18^Ly6G^ cKO), we crossed a mice with a floxed CD18 gene (CD18^fl/fll^) that was generated in our lab (will be described in detail elsewhere) with transgenic mice expressing the Cre recombinase under control of the Ly6G promoter (CD18^wt/wt^ Ly6G^Cre+^). Resulting offspring (CD18^wt/fl^ Ly6G^Cre+^ and CD18^wt/fl^ Ly6G^Cre-^) were fertile and showed no obvious phenotype. These mice were crossed back to CD18^fl/fl^ background yielding CD18^Ly6G^ cKO and CD18^fl/fl Cre-^ mice at expected Mendelian ratios (not shown). All gene-targeted animals were verified by PCR (**Supplementary Figure 1**).

**Figure 1:**
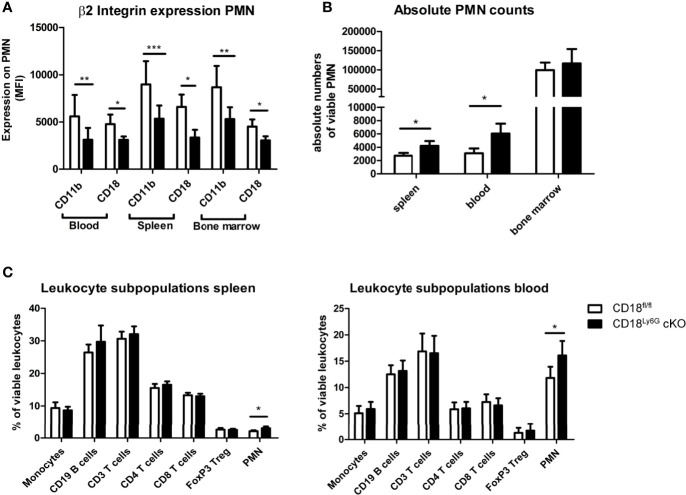
Phenotypical and functional characteristics of CD18^Ly6G^ cKO mice compared to CD18^fl/fl^ mice. We found a significant reduction of β2-integrin surface marker expression (CD11b, CD18) on PMN derived from blood, spleen, and bone marrow **(A)**. Data depict the results of *in-vitro* experiments from n=7-17 mice/genotype. In the same set of experiments we further observed higher absolute and relative counts of PMN in CD18^Ly6G^ cKO mice as compared to CD18^fl/fl^ mice **(B,C)**, whereas the proportions of other leukocyte subpopulations did not differ significantly **(C)** (n=10/genotype). Legend in **(C)** applies to all panels. Statistically significant differences between groups are indicated (*p<0.05, **p<0.005, ***p<0.001).

We could observe a downregulation of CD18 and accordingly of the β2 integrin alpha subunits (CD11a and CD11b) on PMN of CD18^Ly6G^ cKO mice. The extent of downregulation varied between 30-50% compared to CD18^fl/fl^ mice depending on the investigated compartment (blood, spleen or bone marrow) (**Figure 1A**), which is in accordance with the extent of Ly6G *Cre*-mediated downregulation of targeted genes previously shown by Gunzer and coworkers (35). Notably, CD18-reduction was restricted to Ly6G^+^ PMN, and was not observed for CD3^+^ lymphocytes, F4/80^+^ macrophages and Ly6C^+^ monocytic cells, thus confirming the cell-type specific targeting of CD18 (**Supplementary Figures 2, 3**). Absolute PMN counts and relative amounts of PMN in both spleen and blood were found to be slightly higher, whereas PMN counts in the bone marrow did not show significant differences (**Figure 1B**). The percentages of monocytic and lymphocytic cells did not differ significantly between CD18^Ly6G^ cKO mice and CD18^fl/fl^ mice in spleen (**Figure 1C**, left panel) and blood (**Figure 1C**, right panel).

As β2 integrins have also been implicated in the differentiation and in survival signaling of myeloid cells (42), we next investigated whether the PMN-restricted CD18-knockdown affected PMN apoptosis *in-vitro*. Here, we did not find significant differences in the apoptosis of PMN after treatment with GM-CSF or LPS, as assessed by Annexin-V/FVD negative and Annexin-V positive/FVD negative PMN derived from spleens and bone marrow (not shown).

### 3.2 PMN-Specific Knockdown of β2-Integrins Results in an Aggravated Course of IPA

**Figure 2:**
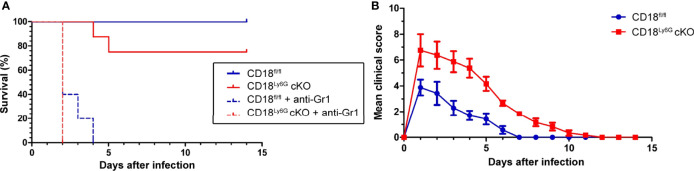
Infection with IPA caused an impaired survival **(A)** and an aggravated course of the disease **(B)** in CD18^Ly6G^ cKO as compared to CD18^fl/fl^ mice. CD18^fl/fl^ and CD18^Ly6G^ cKO mice were infected i.t. with *A fumigatus* (each 10^7^ conidia/mouse) in 2 independent experiments. **(A)** Survival was monitored daily for 2 weeks and is presented in a Kaplan-Meier survival curve. In parallel settings PMN were depleted in some mice *via* injection of an anti-Gr-1 antibody one day before infection. Data show the cumulative results of two independent experiments with a total of 12 (CD18^fl/fl^) and 13 (CD18^Ly6G^ cKO) mice/group. 5 mice/group received an anti-Gr-1 antibody in order to deplete PMN in these mice. All Gr-1 depleted mice died within the first days after IPA infection, whereas all non-depleted CD18^fl/fl^ mice survived. By contrast, some non-depleted CD18^Ly6G^ cKO mice (n=2) deceased within the first week after IPA infection. **(B)** The clinical course of IPA of monitoring was assessed in CD18^fl/fl^ (n=7) and CD18^Ly6G^ cKO mice (n=8) for 14 days. Parameters comprised breathing, reaction to pain overall appearance, hypothermia, strong weight loss, motoric disabilities and apathy (each 0-2).

To assess the relevance of β2 integrins for PMN-specific clearance of pulmonary infection with *A. fumigatus*, we examined the course of disease in CD18^Ly6G^ cKO and CD18^fl/fl^ mice. In some mice an anti-Gr-1 antibody was applied prior to infection with *A. fumigatus* (d0) to deplete PMN as an internal control for the success of infection. As expected, all PMN-depleted mice died during the first days of infection (**Figure 2**), underlining the pivotal role of PMN to limit the spread of *A. fumigatus*. By contrast, all non-depleted CD18^fl/fl^ mice survived infection monitored over 2 weeks, whereas 25% of CD18^Ly6G^ cKO mice died within the first week of infection. This finding is consistent with the observation that clinical signs of IPA infection were more aggravated in case of CD18^Ly6G^ cKO mice in the first days after inoculation. Furthermore, recovery of from clinical symptoms was delayed in CD18^Ly6G^ cKO mice as compared to CD18^fl/fl^ mice (**Figure 2**).

### 3.3 CD18^Ly6G^ cKO Mice Show a Higher Fungal Burden

**Figure 3:**
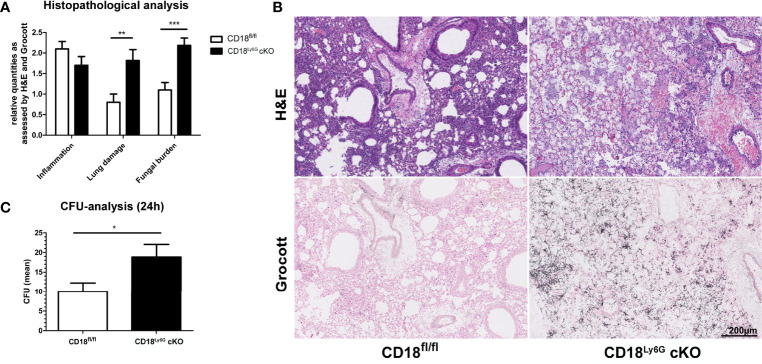
CD18^Ly6G^ cKO mice show a higher pulmonary fungal burden. Histopathological analysis of H&E and Grocott-stained lungs derived from IPA-infected mice 24h upon *A.fumigatus* inoculation revealed a higher fungal burden and a stronger lung damage (i.e., hyaline membranes, fibrin-exudate within the alveoli) in CD18^Ly6G^ cKO mice **(A)**. Cellular inflammation did not show significant genotype-dependent differences. Representative examples of histological analysis are shown in **(B)** (Magnification 10x). Data in **(A)** denote results of histopathological analysis of n=9-10 mice/genotype. We further observed higher CFU counts in serial dilutions of lung homogenates (1:500) after incubation for 24h on Sabouraud-4% Glucose agar plates **(C)**. Data show the mean ± SEM of 6 mice/group. Statistically significant differences between groups are indicated (*p<0.05, **p<0.005, ***p<0.001).

Next, we focused on the course of the early innate immune response towards *A. fumigatus* infection, which is known to be driven by PMN (12). For this, lungs, BALF, and serum of infected mice were analyzed 24h after infection in more detail. Lung homogenates of CD18^Ly6G^ cKO mice showed an enhanced amount of fungal conidia as compared to lungs from CD18^fl/fl^ mice (**Figure 3**). Histopathological analysis confirmed a higher fungal burden and aggravated lung damage in in lungs of CD18^Ly6G^ cKO mice as assessed by Grocott-silver and Hematoxylin & Eosin (H&E) staining. Notably, sprouting of hyphae has only been observed in CD18^Ly6G^ cKO mice. Despite the strong differences in terms of fungal burden, H&E staining of lung tissues showed comparable levels of cellular inflammation, largely irrespective of the genotype (**Figures 3A**). D-Galactomannan-assays revealed that BALF derived from both mice strains contained *A. fumigatus* antigen above detection levels (>5.0), whereas serum analysis showed a higher fungal load in CD18^Ly6G^ cKO mice (mean= 5.8 ± 0.14 vs. 4.7 ± 0.33, p=0.01).

### 3.4 CD18^Ly6G^ cKO Mice Reveal a Decreased Pulmonary Inflammation

**Figure 4:**
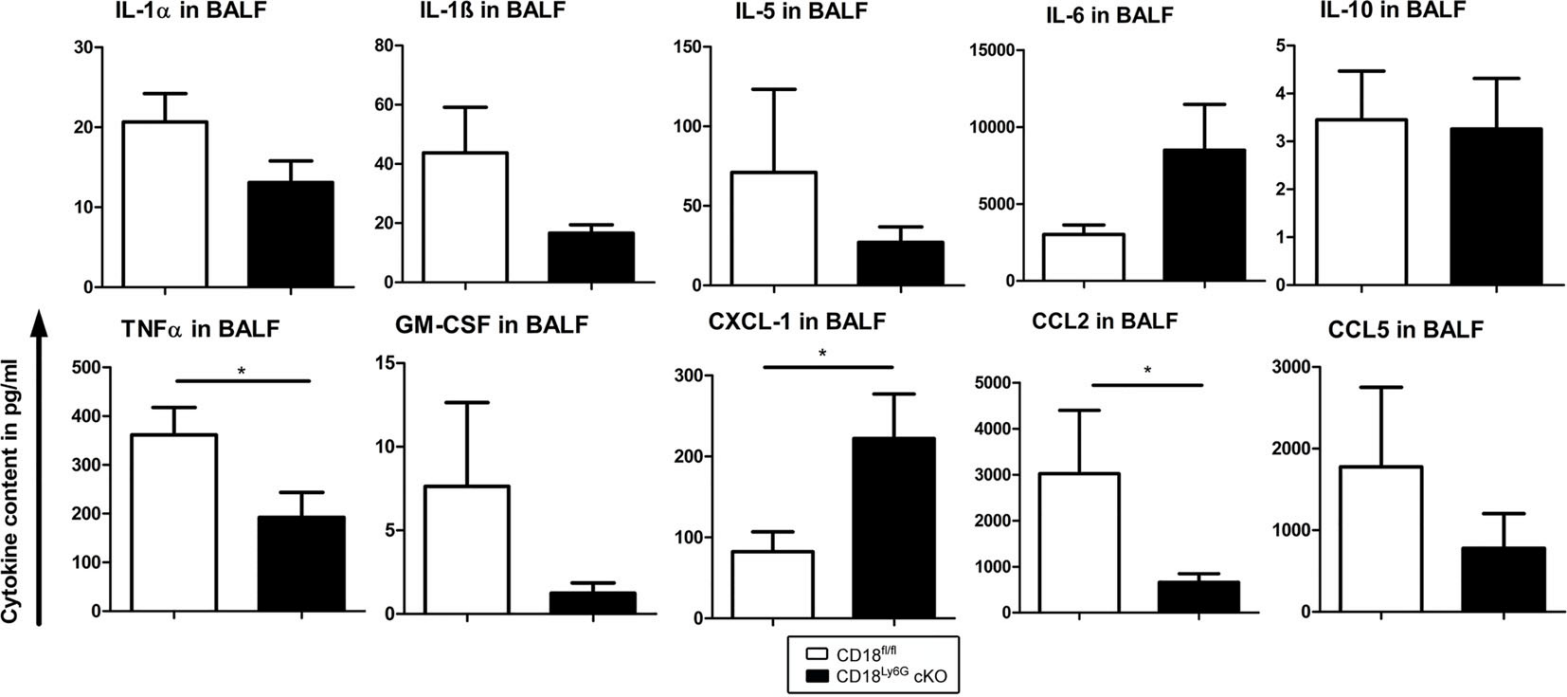
The BAL fluid of *A. fumigatus* infected CD18^Ly6G^ cKO mice contains lower levels of proinflammatory cytokines. CD18^fl/fl^ and CD18^Ly6G^ cKO mice were infected i.t. with *A. fumigatus*. On the next day, mice were euthanized, and cytokines in BAL fluid were analyzed. Data denote the mean ± SEM of 6-10 samples analyzed per group. Statistically significant differences between groups are indicated (*p< 0.05).

In contrast to the increased fungal burden found in lung tissues of CD18^Ly6G^ cKO mice, these mice displayed no significant differences in cellular inflammation as assessed by H&E staining (**Figure 3**). However, as depicted in **Figure 4**, BALF derived from infected CD18^Ly6G^ cKO mice contained lower levels of pro-inflammatory cytokines (TNF-α), and chemokines (CCL2) compared to CD18^fl/fl^ mice, albeit the reduction was below statistical significance in some cases (IL-1α, IL-1β and CCL5). Levels of IL-5, IL-6, IL-10, and GM-CSF were largely comparable. In contrast, BALF obtained from CD18^Ly6G^ cKO mice contained higher levels of the chemokine CXCL-1 known as a relevant chemoattractant for PMN (43).

In contrast, cytokine and chemokine levels in serum were largely comparable between *A. fumigatus* infected CD18^fl/fl^ and CD18^Ly6G^ cKO mice (**Supplementary Figure 5**).

### 3.5 Pulmonary PMN Infiltrates Are Increased in CD18^Ly6G^ cKO Mice Upon IPA

**Figure 5:**
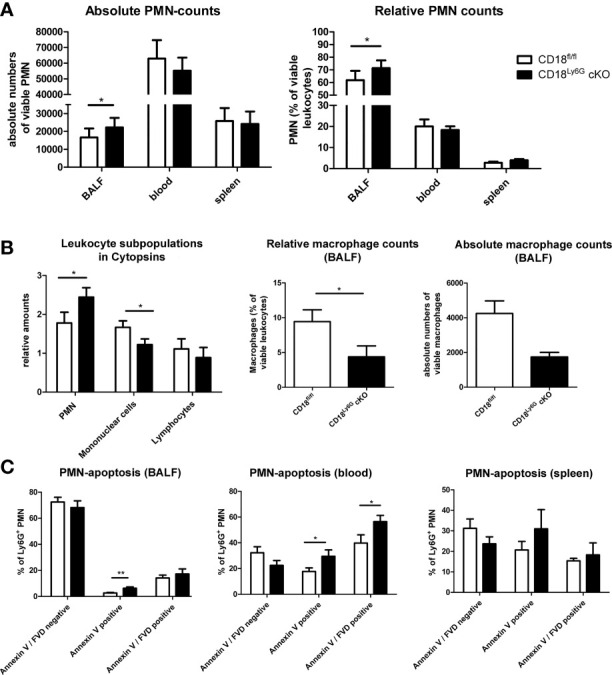
CD18^Ly6G^ cKO mice infected with *A. fumigatus* are characterized by elevated lung infiltration of PMN, but not in spleens and blood (n=10/genotype) **(A)**. Elevated PMN counts have also been found in Cytospins (bars depict the mean ± SEM of n=8 cytospins/genotype; cell infiltration has been assessed using a scoring system; 0=missing - 3=strongest infiltration) **(B)**. Here, we could additionally observe higher numbers of mononuclear cells **(B)**. This is consistent with the finding of higher macrophage counts in the BALF of CD18^fl/fl^ mice as compared to CD18^Ly6G^ cKO mice observed in FACS-analysis **(B)**. Assessment of PMN apoptosis revealed a stronger expression of apoptosis marker Annexin V in PMN derived from BALF and blood of CD18^Ly6G^ cKO mice **(C)**. Bars depict the mean ± SEM of the relative cell counts found in n=8 cytospins/genotype. Legend in A applies to all panels. Statistically significant differences between groups are indicated (*p<0.05)

In accordance with elevated CXCL-1 levels, we observed higher numbers of PMN in the BALF of infected CD18^Ly6G^ cKO as compared to CD18^fl/fl^ mice (**Figure 5A**). In contrast, PMN counts in spleen and blood remained comparable. Higher PMN numbers were also found in cytospin analysis (**Figure 5B**). Here, we could additionally observe lower counts of mononuclear cells in CD18^Ly6G^ cKO mice. Consistent with this observation, results of FACS-analysis revealed lower macrophage counts in the BALF of CD18^Ly6G^ cKO mice (**Figure 5B**).

Notably, a higher frequency of PMN in BALF (**Figure 5C**, left panel) and blood (**Figure 5C**, center panel) obtained from IPA-infected CD18^Ly6G^ cKO mice expressed the early apoptosis marker Annexin-V as compared to CD18^fl/fl^ mice, indicating that CD18-deficient PMN might be more susceptible to apoptosis in response to *A. fumigatus*. In accordance, we observed a higher frequency of Annexin-V positive PMN in spleens of CD18^Ly6G^ cKO mice, albeit the differences here were found to be below statistical significance (**Figure 5C**, right panel).

Besides, our data show that a smaller fraction of PMN derived from BALF of CD18^Ly6G^ cKO mice expressed MHCII (1,6% vs. 4,6% of MHCII^high^ PMN), and CD80 (16.4% vs. 20.1% of CD80^+^ PMN) and showed a lower degree of degranulation as assessed by a low expression of CD62L (88.0% vs. 90.8% CD62L^low^ PMN) than observed for CD18^fl/fl^ mice. BALF-derived PMN of both mice strains expressed the mouse DC marker CD11c at a moderate extent (**Supplementary Figure 6**). Infection-induced *de novo* expression of CD11c by PMN has been reported previously in different mouse infectious disease models (5).

Numbers of PMN, lymphocytes, and monocytes in the peripheral blood of *A. fumigatus* infected mice did not differ in a genotype-dependent manner (**Supplementary Figure 7**). In accordance with our *in-vitro* experiments, we could confirm that the knockdown of CD18 was restricted to Ly6G positive cells (**Supplementary Figure 3**). Similarly, we could observe a knockdown of the corresponding alpha subunits CD11a and CD11b on Ly6G positive PMN of IPA-infected mice (**Supplementary Figure 4**), which is consistent with the physiological role of CD18 as the rate-limiting subunit of β2-integrin surface expression.

### 3.6 Knockdown of CD18 Affects PMN Innate Effector Functions

#### 3.6.1 Phagocytosis

Although PMN were able to infiltrate *A. fumigatus* infected lungs in CD18^Ly6G^ cKO mice, we observed an impaired ability to limit fungal spreading. Hence, we analyzed whether the knockdown of CD18, and thereby β2 integrins, affected the commonly known pathogen-induced immune responses of PMN.

**Figure 6:**
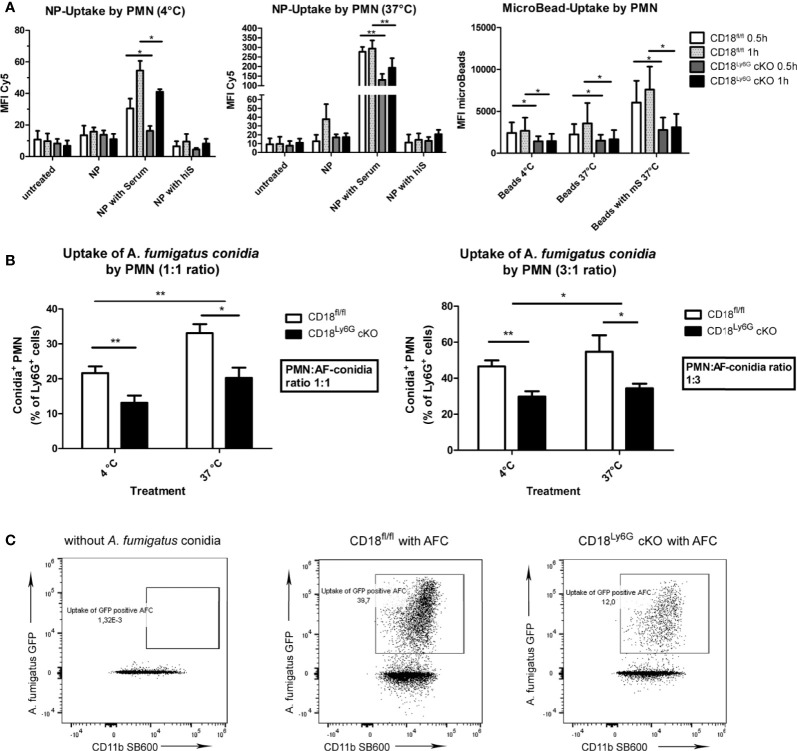
Phagocytosis of inert particles and fungal conidia is less effective in PMN of CD18^Ly6G^ cKO mice. Freshly isolated PMN were co-incubated either with nanoparticles (NP), microBeads **(A)** or with GFP-fluorescent *A. fumigatus* conidia (AFC) at 37°C with indicated ratios **(B)**. Simultaneous co-incubation at 4°C served to differentiate mere adhesion from temperature-dependent binding. After 30 min and 60 min the frequency of either Cy5 positive NP, PE-positive microBeads **(A)**, or GFP-positive PMN **(B)** was determined by flow cytometry. Data represent the mean ± SEM of 3 samples analyzed/group. Exemplary flow cytometry data depicting the diminished uptake of GFP-fluorescent conidia by PMN from CD18^Ly6G^ cKO mice are shown in **(C)**. Statistically significant differences between groups are indicated (*p<0.05, **p<0.005).

As phagocytosis is a major effector mechanism of PMN to clear *A. fumigatus* conidia, we analyzed purified bone marrow-derived Ly6G^+^ PMN to assess potential genotype-dependent differences in this regard. Here, we first investigated the uptake of inert nanoparticles (NP, Ø 50nm) and microBeads (Ø 2µm). In order to dichotomize mere adhesion and energy-dependent uptake we investigated the uptake in parallel settings at 4°C and 37°C. Since MAC-1 has been attributed to serve as a receptor to facilitate complement-opsonized phagocytosis of pathogens we further examined whether the addition of murine serum might enhance the uptake of particles. Heat-inactivated serum which lacks complement activity served as an internal negative control. We could observe for both kinds of particles that their uptake was strongly impaired in case of PMN with a β2 integrin knockdown. This effect was predominantly observed for serum-opsonized particles, indicating that the recognition of complement- opsonized particles might have been diminished in case of CD18 downregulation on PMN (**Figures 6A**). In accordance with the well-known role of MAC-1 (CD11b/CD18) for the binding and uptake of complement-opsonized material, we further observed a significant correlation between CD11b surface marker expression on PMN and the engagement of the aforementioned particles (Pearson´s r: 0.65; p= 0.0007).

Subsequently, we analyzed the phagocytic capacity of PMN after incubation with *A. fumigatus* conidia. Similar to previous experiments with inert particles, we observed a significantly lower phagocytic uptake of *A. fumigatus* conidia by PMN derived from CD18^Ly6G^ cKO mice (**Figure 6B, C**).

#### 3.6.2 NETosis

**Figure 7:**
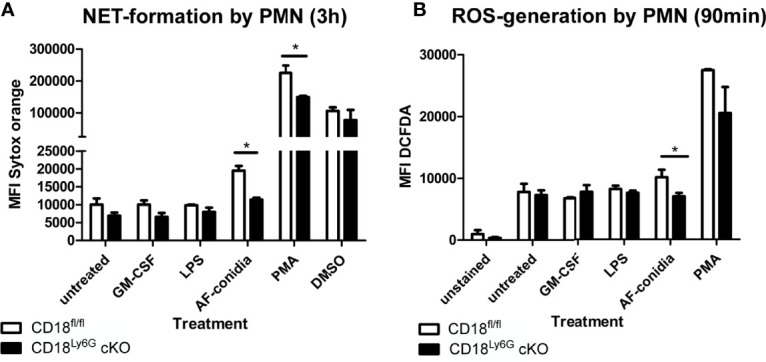
Impaired oxidative and non-oxidative effector functions of CD18-deficient PMN. We could observe that the formation of NET after 3h of incubation **(A)** and ROS-generation **(B)** were significantly lower in PMN derived from CD18^Ly6G^ cKO mice, particularly after stimulation with *A. fumigatus* conidia. Data represent the mean ± SEM of 3 samples analyzed/group. Statistically significant differences between groups are indicated (*p<0.05)

We also investigated the rate of NET-formation of freshly isolated PMN after differential stimulation. Here, we could observe that the formation of NET by PMN derived from CD18^Ly6G^ cKO mice was significantly impaired after treatment with PMA or *A. fumigatus* conidia, as assessed by Sytox orange staining. After treatment with GM-CSF or LPS the differences in the formation of NET by PMN derived from CD18^Ly6G^ cKO vs CD18^fl/fl^ mice were below statistical significance (**Figure 7A**).

#### 3.6.3 ROS-Production

Next, we analyzed the generation of ROS as another important effector mechanism in the innate pathogen defense of PMN. To this end, we incubated freshly isolated PMN with GM-CSF, LPS, PMA or *A. fumigatus* conidia and assessed the generation of ROS *via* DCFDA staining in time intervals of 15min for a total period of 90min. Our results revealed that PMN isolated from CD18^Ly6G^ cKO mice generated significantly lower amounts of ROS after incubation with *A. fumigatus* conidia, suggesting that a CD18 knockdown might impair the ability of PMN to exercise this important effector mechanisms in pathogen-defense (**Figure 7B**). Referring particularly to the time kinetics of ROS-generation we could further observe that the ability to generate ROS was mainly impaired in the course of the first 60min, which indicates that β2 integrins might be implicated in the early generation of ROS (not shown).

#### 3.6.4 Cytokine Secretion

**Figure 8:**
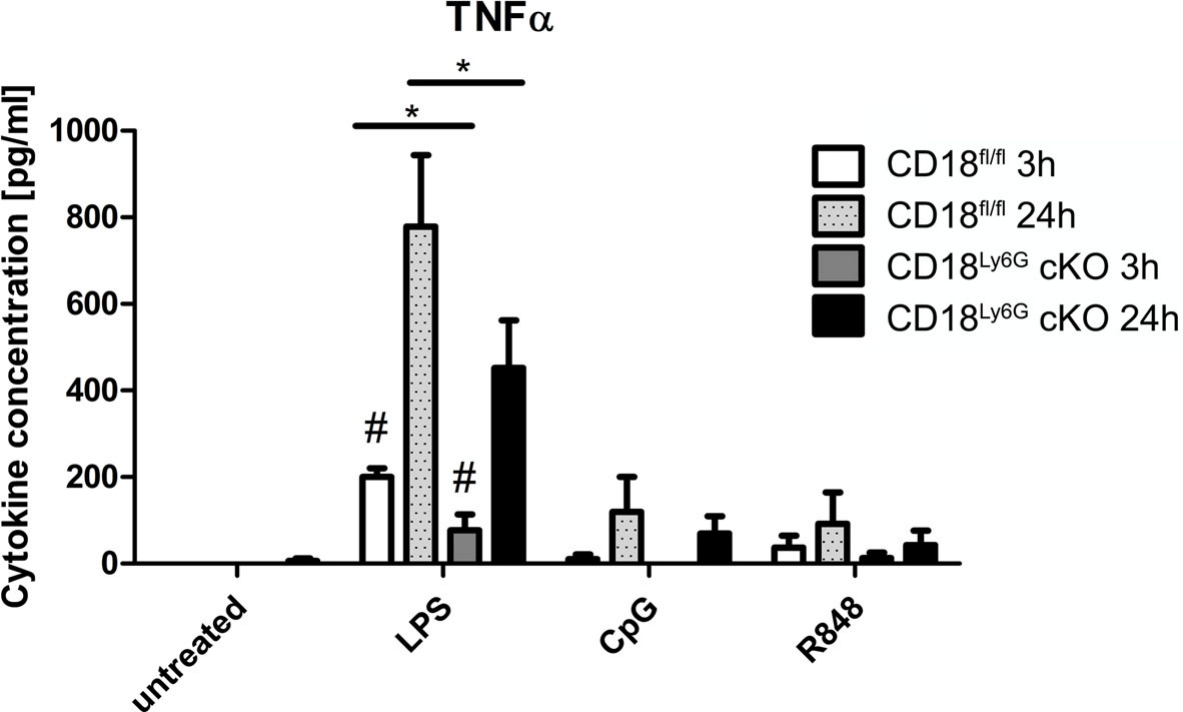
PMN derived from CD18^Ly6G^ cKO mice generate lower amounts of TNF-α after previous stimulation with LPS, CpG or R848. We have purified PMN from CD18^fl/fl^ (n=3) mice and CD18^Ly6G^ cKO (n=3) mice and incubated them for 24h at the indicated conditions. After 3h and 24h supernatants have been taken and were analyzed using a CBA. Results show significantly lower levels of TNF-α in supernatants derived from CD18^Ly6G^ cKO mice. *p<0.05, ^#^p<0.05 when comparing cytokine concentrations from supernatants at 3 vs 24h.

β2 integrins have been found to regulate various signaling pathways in myeloid cells, which modulate the secretion of inflammatory cytokines (27). Hence, we have investigated the generation of cytokines by PMN after *in-vitro* stimulation with GM-CSF, LPS (TLR4 agonist), CpG (TLR9 agonist), and R8/48 (TLR7/8 agonist): Here, we could observe that PMN derived from CD18^Ly6G^ cKO mice generated significantly less amounts of TNF-α upon treatment with LPS (**Figure 8**). On the other hand, we detected significant concentrations of IL-1β, IL-6 and IL-10 upon PMN stimulation, although genotype-dependent differences were largely below statistical significance (**Supplementary Figure 9**). Other cytokines (IL-12, IL-23 or IFN-γ) showed very low concentrations (not shown), suggesting that these cytokines might not be secreted by PMN under the conditions applied. These *in-vitro* data are consistent with our observations from *in-vivo* analysis, showing that BALF and blood derived from CD18^Ly6G^ cKO mice contained lower amounts of TNF-α or IL-1.

#### 3.6.5 RNA-Sequencing Analysis

**Figure 9:**
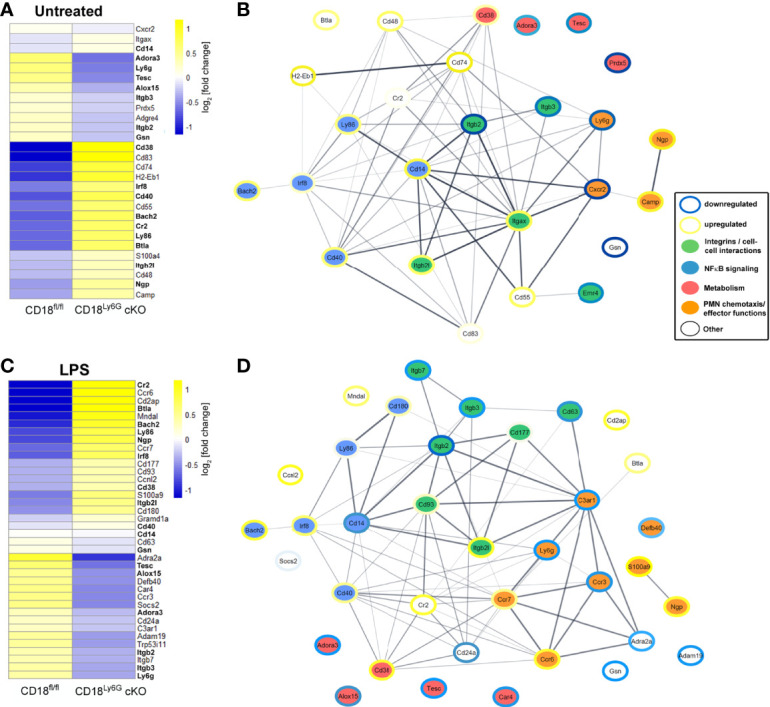
Transcriptomes and functional interaction networks of PMN-associated genes directly after isolation or upon over-night treatment with LPS. PMN were sorted from CD18^fl/fl^ and CD18^Ly6G^ cKO mice (each n=3) and RNA-seq was performed from untreated PMN **(A)** or LPS-treated PMN **(C)**. Expression of indicated PMN-associated genes was analyzed using CLC Genomics Workbench. Genes being differentially regulated both in LPS-treated and freshly isolated PMN are shown in bold **(A, C)**. Predicted interaction networks of the encoded proteins were being visualized using the STRING package in Cytoscape. Genes shown in the interaction networks of untreated PMN **(B)** or LPS-treated PMN **(D)** were categorized into 4 groups affecting either PMN cell-cell interactions, NFκB singaling, PMN metabolism or PMN chemotaxis and PMN effector functions. Colored borders illustrate the degree of the up- or downregulation (log fold change) found for the genes of PMN isolated from CD18^Ly6G^ cKO mice as compared to PMN isolated from CD18^fl/fl^ mice. Legend in **(B, D)**.

Last, we have analyzed the impact of the β2 integrin knockdown on the transcriptome of PMN. To this end, we performed RNA-sequencing analysis of either freshly isolated PMN from CD18^Ly6G^ cKO and CD18^fl/fl^ mice or treated aliquots of isolated PMN over-night with LPS (1µg/ml). This genome-wide gene expression analysis confirmed that both, freshly isolated and LPS-treated CD18-deficient PMN showed a significant downregulation of *Itgb2* and *Ly6G.* Referring to the expression of other integrin genes, we could further observe a downregulation *Itgb3* and *Itgb7*, whereas the CD11c encoding gene *Itgax*, and *Itgb2l* were found to be upregulated in CD18^Ly6G^ cKO PMN. Moreover, RNA-sequencing data revealed that CD18-deficient PMN showed a higher expression of genes implicated in NFκB signaling, such as *CD180*, *Ly86*, *CD14*, *Bach2* or the LPS antagonistic neutrophilic granule protein *Ngp* (44). On the other hand, we could observe a downregulation of genes involved in the inhibition of oxidative effector functions in PMN, such as *S100a9* (45), and a downregulation of genes being implicated in PMN chemotaxis and microbicidal functions (i.e., *Defb40*) in CD18^Ly6G^ cKO PMN (**Figure 9**).

## 4 Discussion

The critical role of β2 integrins for immunological functions is confirmed by the severe immunocompromised state of LAD1 patients, which regularly results in reoccurring invasive bacterial and fungal infections (22, 30). PMN are considered the first line of defense to prevent the spread of inhaled pathogens in the lung (46), and were shown to require β2-integrins for transendothelial migration (24), phagocytosis of opsonized pathogens (21), as well as oxidative, and non-oxidative effector mechanisms (16). Due to the importance of β2-integrins for PMN effector functions and the frequent observation of IPA in LAD1 patients, we aimed to investigate the cell-type specific role of β2 integrins for PMN antifungal effector functions in the early innate immune response to IPA.

Here, we have obtained several key findings that corroborate previous concepts of the pathophysiological role of β2-integrins in the context of severe infections. Our results put these observations into a cell-type specific context and allow insights into the role of CD18 for antifungal effector mechanisms of PMN in the course of IPA, which have not been shown previously.

First, we could observe that the fungal clearance and the early innate immune response in CD18^Ly6G^ cKO mice are significantly impaired. In particular, we found that 24 hours after infection, lungs derived from CD18^Ly6G^ cKO mice showed an enhanced fungal burden and a lower bronchial inflammation as compared to those of CD18^fl/fl^ mice. When PMN are activated upon contact with pathogens and by various danger signals (i.e., the *A. fumigatus* cell wall component ß-glucan), they contribute to the inflammatory immune response in infected tissues by secreting proinflammatory cytokines and chemokines (47). We observed lower levels of innate proinflammatory mediators, such as TNF-α, IL-1α, IL-1ß, and chemokines, like CCL2 and CCL5 in BALF derived from CD18^Ly6G^ cKO mice, suggesting that the knockdown of β2-integrins might have impaired the ability of PMN to generate these inflammatory mediators. Moreover, we observed lower expression levels of markers for PMN degranulation (CD62L) and activation (MHCII, CD80) in PMN derived from CD18^Ly6G^ cKO mice, indicating that the inflammatory signaling pathways in PMN might have also been impaired by CD18 deficiency. In agreement, we found that *A. fumigatus* infected CD18^Ly6G^ cKO mice showed an aggravated course of IPA.

Despite, the significant impairment of the early innate immune response mediated by PMN, the overall survival of *A. fumigatus* infected CD18^Ly6G^ cKO mice was not significantly impaired, suggesting that CD18 despite its pivotal immunoregulatory function might not be critical for the long-term control of IPA or that the residual β2-integrin expression found on PMN of CD18^Ly6G^ cKO mice was sufficient for PMN-mediated pathogen clearance in some mice. Additionally, our results revealed several mechanisms, which may serve to compensate for the impaired effector functions of PMN in CD18^Ly6G^ cKO mice upon infection: Particularly, we could observe a higher level of the PMN-attracting chemokine CXCL-1 in BALF obtained from CD18^Ly6G^ cKO mice. Accordingly, a significantly higher bronchial infiltration by PMN has been found in these mice. These findings were unexpected as β2 integrins were reported to be necessary for the firm adhesion of PMN to vessel endothelium as a prerequisite of PMN migration into the extravascular space (48). In this regard, it has been suggested, that the requirement of CD18 for PMN infiltration might depend on the type of pathogen used in CD18^−/−^ mice (49) and the disease specific context investigated (50). Also, it has been suggested by Mackarel and coworkers, that PMN migration into inflamed lungs might occur either *via* a CD18-dependent or CD18-independent route, which is selected depending on whether inflammation is acute or chronic (51). In particular, Mizgerd and coworkers reported that intratracheal instillation with *E. coli* or *Ps. aeruginosa* resulted in a limited pulmonary PMN-infiltration, whereas infection with *S. pneumonia* yielded a stronger PMN-infiltration in a CD18-independent manner (49). These observations are consistent with previous reports, which have demonstrated that CD11b^−/−^mice infected with either *S. pneumoniae* (52) or *A. fumigatus* (5), showed an elevated PMN infiltration 24 hours upon infection. However, also in these disease models a higher pulmonary burden and a diminished cellular inflammation have been reported. Similarly, a stronger pulmonary infiltration by PMN has been observed in LAD1 patients suffering from pneumonia (53), suggesting that MAC-1 might not be essential for PMN migration. Rather β2 integrin deficiency may be compensated by other adhesion receptors in a disease specific manner (22). In this context, some studies have reported that LFA-1 (CD11a/CD18) may play a dominant role for transendothelial migration of PMN (51, 54). Our results could however not reveal an upregulation of CD11a on PMN, but rather showed a significant downregulation of CD11a in the context of CD18-deficiency. This is in line with the physiological regulation of β2-integrins on PMN. In particular, the downregulation of CD18 in our knock-out mouse model limits the amount of intracellular available CD18 protein and thus heterodimerization with the corresponding alpha subunits on the cell surface is also being restricted, resulting in lower expression levels of LFA-1 (CD11a/CD18) and MAC-1 (CD11b/CD18) on PMN. On the other hand, RNA-sequencing results indicated that other β2 integrin-associated genes (such as the CD11c coding *Itgax* or *Itgb2l*) and genes coding for chemokine receptors (i.e., *Ccr7*) might be upregulated in PMN isolated from CD18^Ly6G^ cKO mice potentially revealing another compensatory mechanism (**Figure 9**). Altogether, our results suggest that the knockdown of β2 integrins (LFA-1, MAC-1) might not significantly impair the pulmonary migration of PMN. However, when interpreting the results of our analysis, it has to be taken into account that a significant residual expression of CD18 was still being observed on PMN, which might allow for the CD18-dependent migration of PMN into inflamed pulmonary tissue.

Furthermore, we could demonstrate that PMN isolated from CD18^Ly6G^ cKO mice showed an impaired phagocytic activity towards opsonized *A. fumigatus* conidia and inert particles as compared to CD18^fl/fl^ PMN, which is consistent with the enhanced pulmonary fungal burden found in *A. fumigatus* infected CD18^Ly6G^ cKO mice. This finding is in agreement with previous observations that MAC-1 is required in human PMN to recognize ß-glucan containing structures (55), such as *A. fumigatus* conidia, and thus to kill conidia by phagocytic uptake (21, 56).

In contrast to small-sized conidia, recognition of *A. fumigatus* hyphae has been largely attributed to IgG and Fcγ receptors (21). However, cross-linking of MAC-1 upon pathogen-recognition, also results in an NADPH-oxidase-dependent oxidative burst by PMN, which is required for an efficient fungal clearance of both *A. fumigatus* conidia and hyphae (19, 21, 57–59). Oxidative burst protects against invasive fungal infections, because it induces apoptosis-like cell death in fungal conidia (60) and contributes to the formation of NET (61, 62). The latter is considered a mechanism of extracellular killing of hyphae, being too large to be phagocytosed (63). The proposed role of MAC-1 for the induction of ROS and the formation of NET by PMN upon incubation with *A. fumigatus* conidia indicates that both antifungal killing-mechanisms might be impaired in CD18^Ly6G^ cKO mice. These findings are again consistent with previous reports, which demonstrated that CD11b^−/−^ mice displayed an attenuated PMN killing activity and increased fungal burdens in a mouse model of candidiasis, thus underpinning the pivotal role of β2 integrins for antifungal effector mechanisms (64), such as CR3-mediated phagocytosis, NETosis (65) and ROS-generation (20, 66). Interestingly, Yakubenko and coworkers have more recently observed that neutrophil oxidative burst might further contribute to a positive feedback loop with β2 integrins by enhancing the affinity of MAC-1 ligands to MAC-1 on macrophages, thus stimulating their migratory activity (67).

Next to the direct cytotoxic effects exerted by PMN, some studies reported that the engagement of MAC-1 with extracellular pathogens also promotes proinflammatory signaling pathways in PMN *via* activation of members of the NF-κB transcription factor family, thus yielding an elevated production of proinflammatory cytokines such as IL-1 and TNF-α (68, 69). In agreement we observed that a knockdown of β2 integrins impaired the secretion of TNF-α. Moreover, it has been found that CD11b facilitates TLR-4 mediated proinflammatory immune responses by promoting MyD88 signaling pathways (27). Hence, the impaired induction of an inflammatory milieu in the lungs of CD18^Ly6G^ cKO mice might be a consequence of the attenuated PMN activation, resulting from a reduced activity of CD18-deficient PMN to recognize and phagocytose *A. fumigatus* conidia and to promote TLR-4-induced signaling pathways.

Besides the diminished levels of proinflammatory cytokines found in BALF obtained from CD18^Ly6G^ cKO mice, we could also observe lower levels of macrophage attracting chemokines CCL2 and CCL5 therein. CCL5 is known to attract many leukocyte populations, such as macrophages and PMN (70–72). Early in the course of inhalative inflammation, CCL5 is generated by various activated cell types, including airway epithelial cells (73) or lung fibroblasts (74). Moreover, *A. fumigatus* was reported to induce CCL5 in platelets (75), and activated PMN were demonstrated to produce CCL5 when incubated with *Toxoplasma gondii* (76). Therefore, it is conceivable that a reduced level of β2 integrins on PMN might impair their ability to generate CCL5.

CCL2, also known as monocyte chemoattractant protein (MCP)-1 is an important regulator of monocyte and macrophage trafficking during infection and in the presence of inflammation (77–79). CCL2 is generated by pulmonary epithelium (80), endothelial cells (81), fibroblasts and T cells upon induction with inflammatory stimuli such as LPS or IFN-γ (82). Notably, also PMN contribute to CCL2 generation, which can be induced upon TLR2-/TLR4-activation (82, 83). CCL2 mainly serves as a chemoattractant for monocytes and macrophages (82, 84–86). Beyond its role as a monocyte chemoattractant CCL2 has been implicated in various molecular and cellular processes impacting myeloid cell functions and their response to pathogens. In particular, it has been shown that CCL2 induces β2 integrin expression on monocytes, thus promoting their migration into inflamed tissues (82, 87) Moreover an enhanced survival and an augmented generation of proinflammatory cytokines by CD11b^+^ cells has been demonstrated upon CCL2 treatment (88). CCL2 treatment has further been shown to induce respiratory burst in monocytes, thus contributing to myeloid cell effector functions in response to pathogens (82, 89). In agreement, increased CCL2 levels have been reported to improve the clearance of pathogens and the survival of *S. pneumonia* infected mice (90). These studies are consistent with our observations that CD18^fl/fl^ mice show higher levels of CCL2, a lower fungal burden and a stronger pulmonary infiltration with macrophages, which might exert critical antifungal effector mechanisms in the early innate response to *A. fumigatus* infection (90). Due to impaired signaling in CD18-deficient PMN it also seems conceivable, that PMN might generate less CCL2 and CCL5 in CD18^Ly6G^ cKO mice. However, as for the multiple sources of these chemokines, further studies are required to elucidate which cell types are responsible for the different concentrations of CCL2 and CCL5 in the lungs of *A. fumigatus* infected CD18^Ly6G^ cKO mice and which cells are most likely to be attracted in response to these chemokines.

In addition to migration, pathogen recognition/phagocytosis, and the regulation of cell signaling, MAC-1 has also been implicated in myeloid cell survival. Referring particularly to PMN apoptosis, we could observe that PMN derived from *A. fumigatus* infected CD18^Ly6G^ cKO mice showed a stronger expression of apoptosis marker Annexin-V, suggesting that a knockdown of β2 integrins might impair PMN survival. This is in contrast to previous *in-vitro* experiments from Coxon and coworkers, which suggested that CD11b contributes to PMN survival, as CD11b^−/−^ PMN isolated from the peritoneum after injection of thioglycollate were characterized by lower apoptosis than their wild-type counterparts (42). However, the contribution of MAC-1 signaling to apoptosis of activated PMN is still subject to controversial discussion. For example, another report by Zhang *et al.* showed that phagocytosis of pathogens by PMN promoted apoptosis of the latter, which was associated with the induction of reactive oxygen species and was enhanced by TNF-α (91). In contrast, CD11b^−/−^ PMN were not found to undergo phagocytosis-induced apoptosis. Similar findings were reported for human PMN (92). On the contrary, Yan and coworkers showed that antibody-mediated blockade of β2 integrins on human PMN elevated apoptosis after their activation by TNF-α or microbial stimuli (93). Since CD18^Ly6G^ cKO mice only showed a moderate, PMN-restricted, LAD1 phenotype with a residual β2-integrin expression on PMN it seems conceivable that apoptosis may not have been significantly impaired, whereas the same moderate reduction of CD18 on PMN might yet affect other PMN effector functions, as well as the overall course of the disease. Hence, further studies are warranted to elucidate the exact role of MAC-1 on PMN viability during pathogen control.

Although our study focused on the role of β2 integrins for PMN effector mechanisms during early innate immune responses towards inhalative infection with *A. fumigatus*, it is likely that a knockdown of β2 integrins might not only impair PMN functions but may also modulate their interaction with other immune cells implicated in IPA-resolution, such as DC (94), macrophages, lymphocytes or eosinophils (95, 96). Here, a report by Park and coworkers could show that PMN contribute to pulmonary infiltration of CD11b^+^ conventional DC in IPA by activating CD11b^+^ DC *via* DC-SIGN (94). This C-type lectin receptor expressed by DC and macrophages mediates the phagocytic uptake of *A. fumigatus* conidia (97) and engages with PMN-bound MAC-1 upon DC-PMN interaction (98). Hence, MAC-1 on PMN may further contribute to the activation of infiltrating DC, which produce IL-12 and IL-23, thus inducing Th_1_ immunity in IPA (99). Notably, IL-23 has also been reported to stimulate IL-17 production in PMN, and IL-17 induced ROS production by PMN (100), contributing to the killing of *A. fumigatus* conidia and hyphae. Thus, the diminished expression of CD18 on PMN might further impair their interaction with DC, contributing to an impaired antifungal immune response in CD18^Ly6G^ cKO mice. However, we could not find significant differences neither in IL-17 nor IL-23 secretion in BALF and serum. An important limitation of our experiments is the residual expression of β2-integrins on PMN derived from CD18^dLy6G^ mice which may only result in a moderate impairment of PMN effector. On first sight, an adoptive transfer of PMN from CD11b^-/-^ mice into infected WT mice after depletion of WT PMN may be suitable to give more comprehensive insights into the PMN-specific role of β2-integrins during invasive *A. fumigatus* infections and exclude compensatory effects that might result from an intermediate PMN phenotype. In this context it would also be interesting to evaluate whether the addition of WT PMN into CD18^Ly6G^ cKO mice might reverse a severe course of the disease. However, such adoptive transfer studies might be subject to methodological bias, including the rather short life span of PMN in general, the influence of β2 integrins on PMN viability and the possibility of artificial PMN activation during adoptive transfer procedures. In conclusion, our results demonstrate, that the PMN-specific downregulation of CD18 allows for a distinct cell-type specific analysis of the role of β2 integrins for PMN effector functions, PMN signaling, survival and the role of β2 integrins as regulators within the immune cell network (47). We could further show that CD18 deficiency on PMN particularly affects the early course of IPA, which might be attributed to the critical role of MAC−1 for PMN antifungal effector mechanisms, such as phagocytosis and ROS-generation (5). However, we cannot rule out that the CD18-knockdown might cause additional unrecognized effects in PMN effector functions, such as the release of primary granules or MPO-activity contributing to the clearance of *A. fumigatus* or that residual CD18 expression might compensate some impaired effector functions. Taking into account that previous PMN-specific knock-out models, such as the Syk^fl/-^ MRP8Cre^Tg^ mice reported by van Ziffle and Lowell (101), also showed a residual expression of the targeted proteins on PMN, further work is necessary to generate knock-out models which might allow for a complete knock-out of β2-integrins on PMN. Also, additional studies will be necessary to elucidate the long-term course of IPA in CD18^Ly6G^ cKO mice with regard to the interplay of PMN with DC, the efficacy of adaptive immune responses and the contribution of chemokines such as CCL2 and CCL5.

## Data Availability Statement

The datasets presented in this study can be found in online repositories. The names of the repository/repositories and accession number(s) can be found below: Gene Expression Omnibus, GSE195444.

## Ethics Statement

The animal study was reviewed and approved by the National State Investigation Office Rhineland-Palatinate, Approval ID: 23177-07/G16-1-020.

## Author Contributions

MH designed methods for *in-vitro* and *ex-vivo* experiments, carried out experiments, performed image analysis, carried out data analysis, calculated statistics, designed and generated figures, compiled tables, and wrote the manuscript. MBro designed experiments, designed methods for *in-vitro* experiments, performed image analyses, edited and designed figures and tables, and helped writing the manuscript. FR, DT and MK helped to design and carry out the *in-vivo* experiments, helped writing the manuscript and edited the manuscript. MK and ES helped carrying out the experiments. SG and MR helped designing the experiments, as well as writing and editing the manuscript. MBed (Monika Bednarczyk) generated the CD18^fl/fl^ and CD18^Ly6G^ cKO mouse. MG helped generating the CD18^Ly6G^ cKO mouse strain, provided C57BL/6-*Ly6g*(tm2621(Cre-tdTomato)Arte mice and AF strains (ATCC 46645 and Afs148) and helped editing the manuscript. All authors contributed to the article and approved the submitted version.

## Funding

MH was supported by the Clinician Scientist Fellowship “TransMed Jumpstart Program: 2019_A72” supported by the Else Kröner Fresenius Foundation and by an intramural research funding of the UMC Mainz. MR is funded by the TRR156 KS01 and SFB1066 TPB10. SG is funded by the DFG (TRR156, B11; SFB1066, B04).

## Conflict of Interest

The authors declare that the research was conducted in the absence of any commercial or financial relationships that could be construed as a potential conflict of interest.

## Publisher’s Note

All claims expressed in this article are solely those of the authors and do not necessarily represent those of their affiliated organizations, or those of the publisher, the editors and the reviewers. Any product that may be evaluated in this article, or claim that may be made by its manufacturer, is not guaranteed or endorsed by the publisher.
