# Supplementary material for: Neutrophil-Specific Knockdown of β2 Integrins Impairs Antifungal Effector Functions and Aggravates the Course of Invasive Pulmonal Aspergillosis
Source: Front Immunol. 2022 Jun 6;13:823121. doi: 10.3389/fimmu.2022.823121 (PMC9207500; doi:10.3389/fimmu.2022.823121)
Supplement: Supplementary file 1 [file DataSheet_1.pdf]

Supplementary Figures and Tables

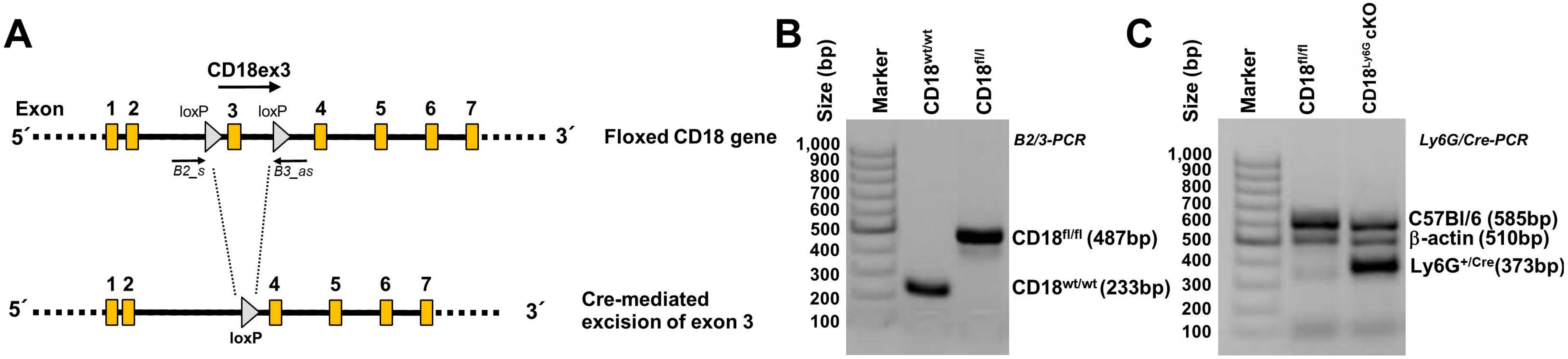

Supplementary Figure 1: Recombination strategy and verification of gene amplification products via PCR. (A) depicts the recombination strategy for the generation of CD18$^{fl/fl}$ mouse. The targeting vector encoded CD18 exon 3 was flanked by loxP sites. After Cre mediated excision, exon 3 of CD18 has been excided from the genome, resulting in a complete CD18 deletion in the specific cell type (here: Ly6G-specific CD18 deletion). The primer pairs used to verify successful Cre-mediated excision of exon 3 are shown in the upper panel (B2_s and B3_as). **(B)** depicts the results from a representative B2/3-PCR (PCR-1). Using the primer pair B2/3 a single 487bp product was amplified in case of CD18$^{fl/fl}$ homozygote and a single 233bp product in case of the CD18$^{wt/wt}$ mice. **(C)** shows representative PCR-results for a Ly6G/Cre-PCR (PCR-2). Using the primer pairs previously described by Hasenberg and coworkers and a primer pair detecting ß-actin, a single 373bp product was amplified in case of a successful knock-in (Ly6G^{Cre+}) and a single 585bp product in case of Cre negative wild-type mice (C57Bl/6). Mice with a floxed CD18 gene as determined in PCR-1 and a Ly6G^{Cre+} genotype in PCR-2 were therefore being classified as CD18^{Ly6G} cKO mice. Whereas mice without the respective knock-in-band in PCR-2 were classified as CD18$^{fl/fl}$.

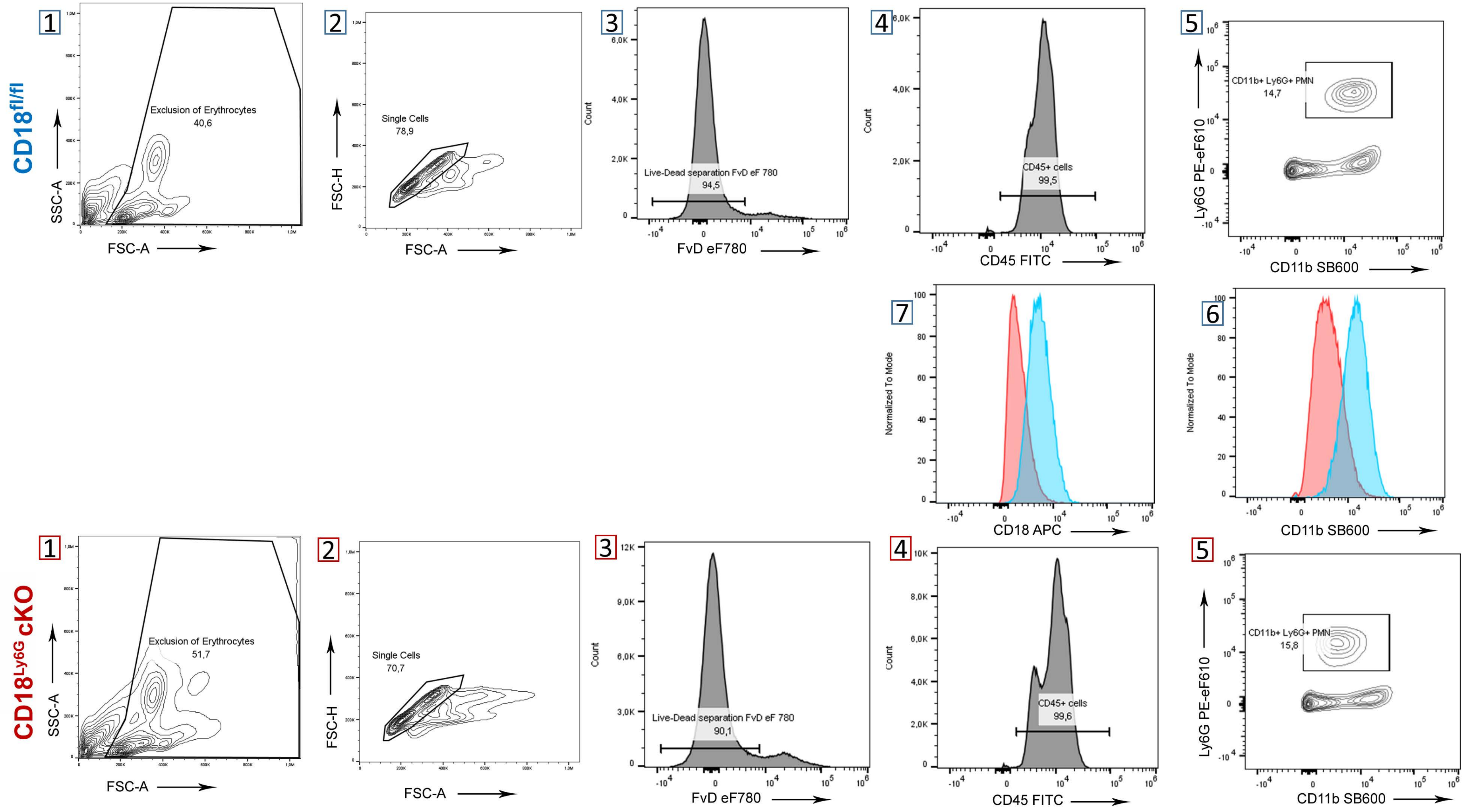

Supplementary Figure 2: Representative example of the gating strategy applied in the *in-vivo* experiments. Gating strategy comprises exclusion of erythrocytes (1), doublets (2) and non-viable cells (3). Subsequently we have identified PMN as CD45+ (4), Ly6G+ and CD11b+ cells (5). Data reveal a significant downregulation of CD11b (6) and CD18 (7) on PMN derived from $CD18^{Ly6G}$ cKO mice (red) as compared to $CD18^{fl/fl}$ mice (blue).

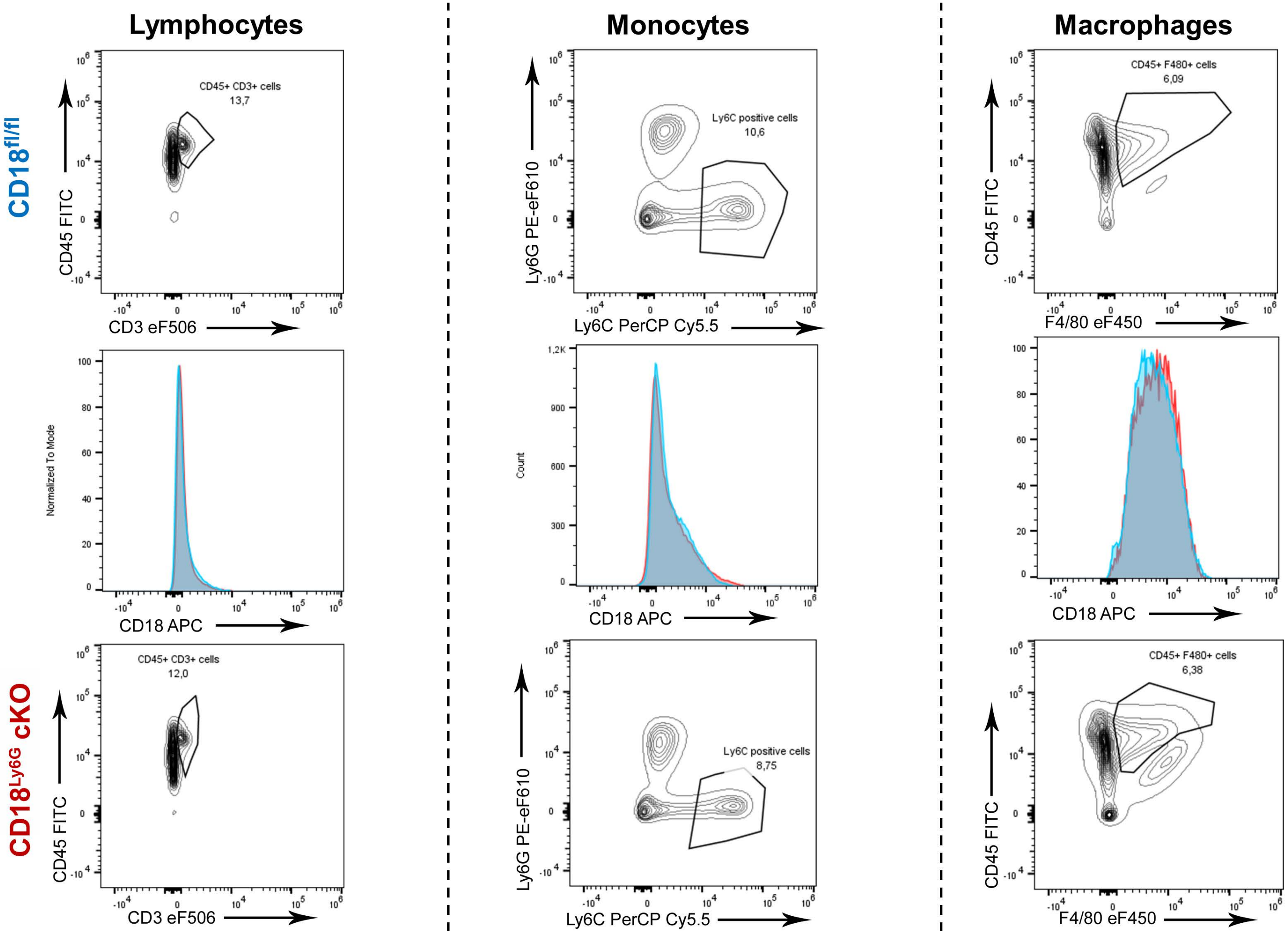

Supplementary Figure 3: Downregulation of CD18 is restricted to PMN. Gating for $CD3^{+}$ lymphocytes (left column), $Ly6C^{high}$ $Ly6G^{low}$ monocytes (center column) and $F4/80^{+}$ macrophages (right column) revealed that these leukocyte subpopulations have not been affected by CD18-downregulation (histograms, blue: $CD18^{fl/fl}$ mice; red: $CD18^{Ly6G}$ cKO mice).

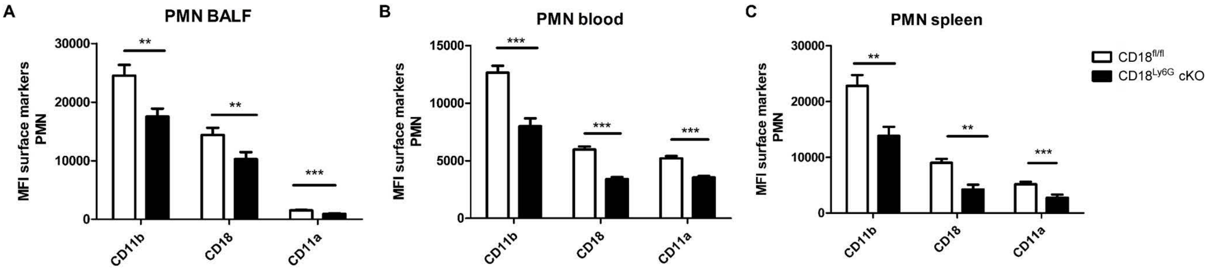

Supplementary Figure 4: β2-integrin subunit surface marker expression on PMN from BALF (A), blood (B) or spleens (C) of IPA-infected CD18$^{fl/fl}$ and CD18^{Ly6G} cKO mice. We could observe a marked downregulation (30-50%) of all subunits on PMN derived from these compartments in CD18^{Ly6G} cKO mice. Data represent the mean ± SEM of 6-10 samples analyzed /group during *in-vivo* experiments. Statistically significant differences between groups are indicated (**p<0.005, ***p<0.001).

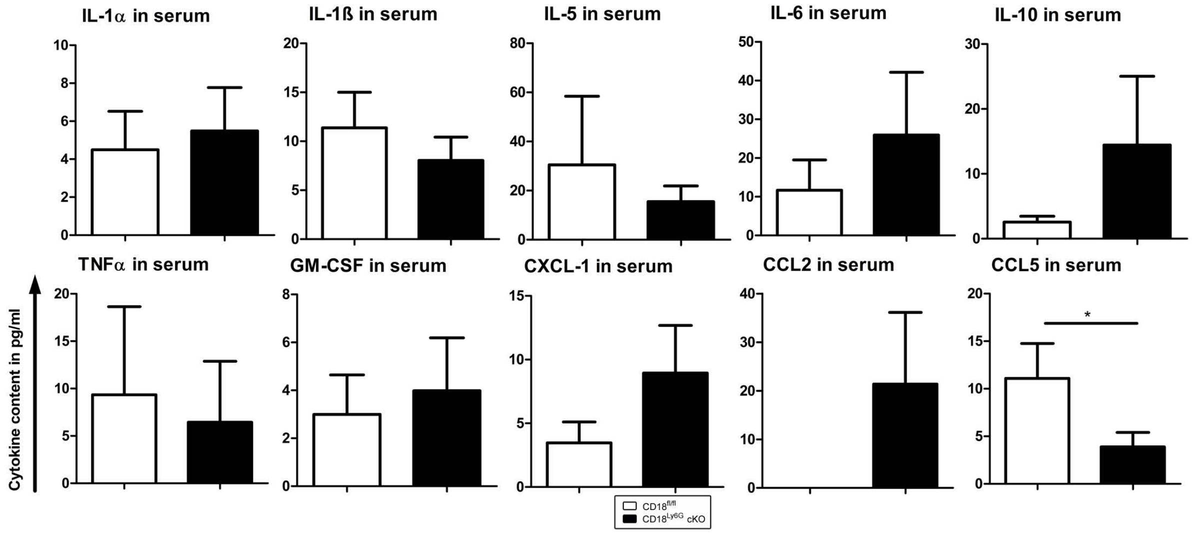

Supplementary Figure 5: Cytokine measurements from serum of IPA-infected CD18$^{fl/fl}$ and CD18^{Ly6G} cKO mice. Despite a stronger expression of CCL5 in serum of CD18$^{fl/fl}$ mice, results did not reveal significant differences between the different genotypes. Data denote the mean ± SEM of 6-10 samples analyzed per group. Statistically significant differences between groups are indicated (*p<0.05).

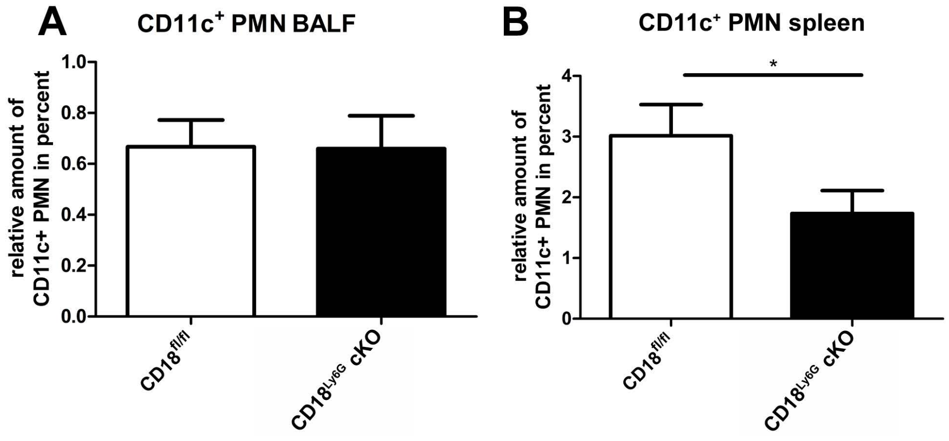

Supplementary Figure 6: Share of $CD11c^+$ PMN among all PMN found in spleens or BALF of $CD18^{fl/fl}$ and $CD18^{Ly6G}$ cKO mice, respectively. In accordance with previous reports, we found a small share of PMN with a strong expression of CD11c. CD11c expression among splenic PMN derived from $CD18^{fl/fl}$ mice was higher as compared to $CD18^{Ly6G}$ cKO mice. No statistically significant differences in CD11c expression on BALF-derived PMN have been observed. Data show the mean ± SEM of 6-10 samples analyzed per group. Statistically significant differences between groups are indicated (*$p<0.05$).

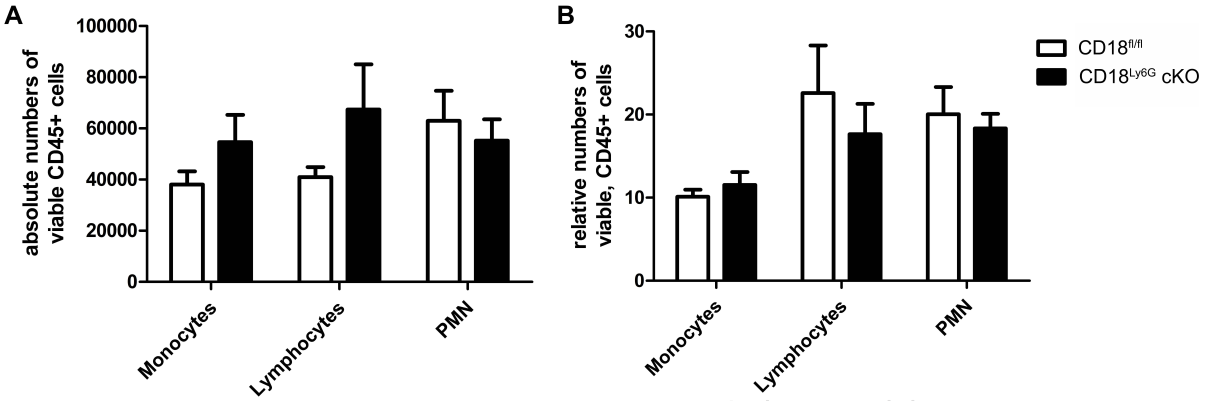

Supplementary Figure 7. Relative and absolute numbers of leukocyte subpopulations analyzed in peripheral blood. No significant differences between the relevant cell populations have been observed. However, as for absolute cell counts, we found a moderate leukocytosis in $CD18^{Ly6G}$ cKO mice. Monocytes were classified as $Ly6C^{high}$ and $Ly6G^-$, whereas PMN were defined as $Ly6G^{high}$ and $Ly6C^-$. Lymphocytes denote all $CD3^+$ T cells. Data represent the mean ± SEM of 6-10 samples analyzed /group during *in-vivo* experiments.

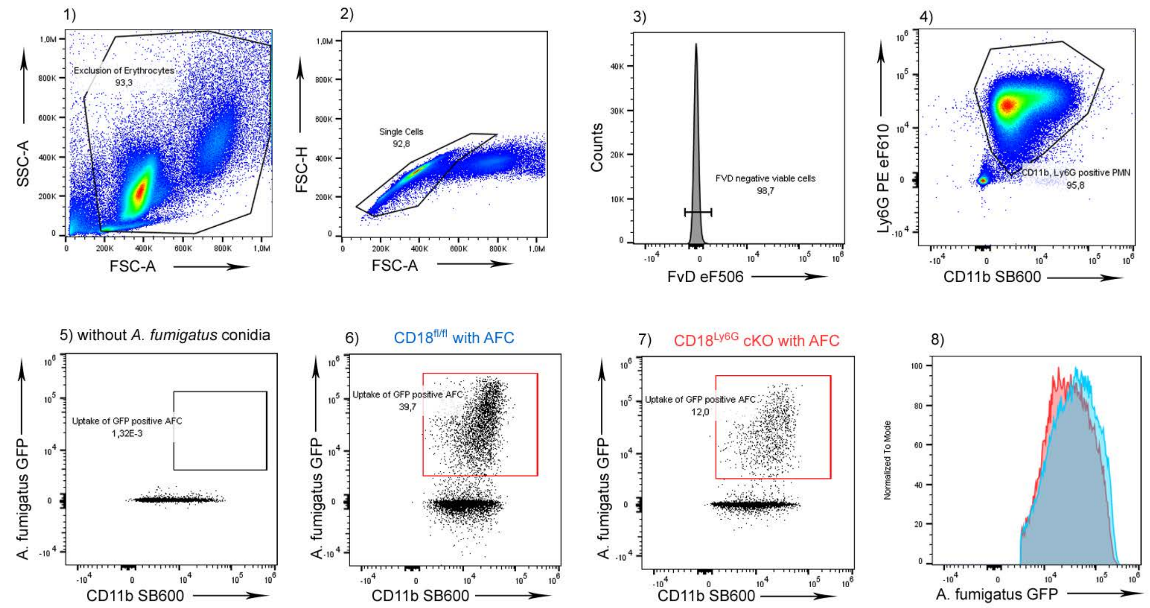

Supplementary Figure 8: Gating strategy applied in the experiments analyzing the uptake of GFP-fluorescent A. *fumigatus conidia* (AFC) by PMN. Here, we could observe a diminished uptake of GFP-fluorescent conidia by PMN isolated from CD18^{Ly6G} cKO mice as compared to PMN isolated from CD18$^{fl/fl}$ mice (red vs. blue histograms in 8).

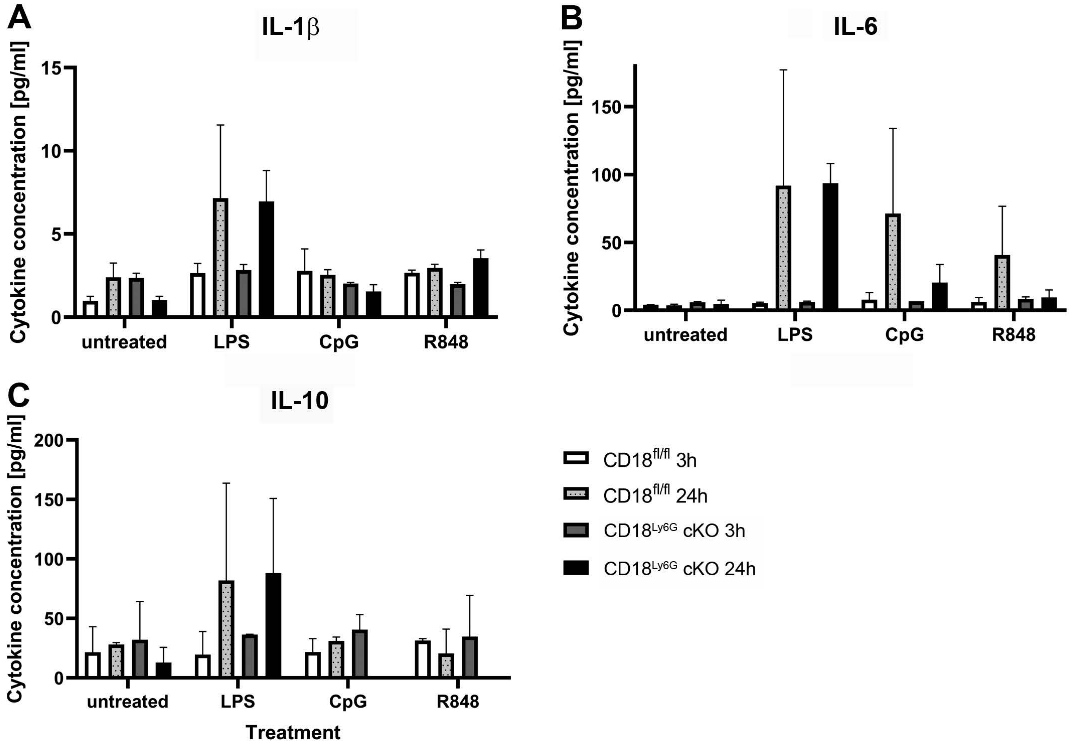

Supplementary Figure 9: Results from in-vitro cytokine analysis of PMN isolated from CD18$^{fl/fl}$ and CD18^{Ly6G} cKO mice after 3h and 24h of differential treatment. We have purified PMN from CD18$^{fl/fl}$ (n=3) mice and CD18^{Ly6G} cKO (n=3) mice and incubated them for 24h at the indicated conditions. After 3h and 24h supernatants have been taken and were analyzed using a CBA. We could detect no significant genotype-dependent differences in cytokine levels of IL-1β, IL-6 or IL-10 in supernatants. Bar charts denote the mean + SEM of cytokine measurements from n=3 experiments.
